# Wnt signaling modulates mechanotransduction in the epidermis to drive hair follicle regeneration

**DOI:** 10.1126/sciadv.adq0638

**Published:** 2025-02-19

**Authors:** Allen S. W. Oak, Amrit Bagchi, Matthew J. Brukman, Joshua Toth, Jamie Ford, Ying Zheng, Arben Nace, Ruifeng Yang, Jen-Chih Hsieh, James E. Hayden, Gordon Ruthel, Anisa Ray, Elaine Kim, Vivek B. Shenoy, George Cotsarelis

**Affiliations:** ^1^ Department of Dermatology, Perelman School of Medicine, University of Pennsylvania, Philadelphia, PA, USA.; ^2^ Department of Materials Science and Engineering, University of Pennsylvania, Philadelphia, PA, USA.; ^3^ Center for Engineering Mechanobiology, University of Pennsylvania, Philadelphia, PA, USA.; ^4^ Singh Center for Nanotechnology, University of Pennsylvania, Philadelphia, PA, USA.; ^5^ Wistar Institute, Philadelphia, PA, USA.; ^6^ Department of Pathobiology, School of Veterinary Medicine, University of Pennsylvania, Philadelphia, PA, USA.

## Abstract

Most wounds form scars without hair follicles. However, in the wound-induced hair neogenesis (WIHN) model of skin regeneration, wounds regenerate hair follicles if tissue rigidity is optimal. Although WIHN depends on Wnt signaling, whether Wnt performs a mechanoregulatory role that contributes to regeneration remains uncharacterized. Here, we demonstrate that Wnt signaling affects mechanosensitivity at both cellular and tissue levels to drive WIHN. Atomic force microscopy revealed an attenuated substrate rigidity response in epidermal but not dermal cells of healing wounds. Super-resolution microscopy and nanoneedle probing of intracellular compartments in live human keratinocytes revealed that Wnt-induced chromatin remodeling triggers a 10-fold drop in nuclear rigidity without jeopardizing the nucleocytoskeletal mechanical coupling. Mechanistically, Wnt signaling orchestrated a massive reorganization of actin architecture and recruited adherens junctions to generate a mechanical syncytium—a cohesive contractile unit with superior capacity for force coordination and collective durotaxis. Collectively, our findings unveil Wnt signaling’s mechanoregulatory role that manipulates the machinery of mechanotransduction to drive regeneration.

## INTRODUCTION

Mammals exhibit limited regenerative capabilities, often leading to fibrotic wound healing (*1*). This profibrotic response is driven by soluble mediators and exacerbated by the stiff mechanical properties of the wound environment, including that of the extracellular matrix (ECM) (*2*–*4*).

Mechanical stimuli, including matrix rigidity, are converted to biochemical signals through mechanotransduction, which elevates cytoskeletal rigidity through actomyosin (actin-myosin complex) recruitment and activates mechanosensitive pathways to broadly influence cell behaviors (*5*). The proportional relationship between environmental (i.e., substrate) rigidity and cellular mechanotransduction is called the substrate rigidity response (*5*, *6*). Increased mechanotransduction in wounds generates a robust profibrotic response (*2*, *3*).

Wound-induced hair neogenesis (WIHN), characterized by regeneration of hair follicles in large wounds, is an established model of mammalian regeneration (*4*, *7*–*9*). We know that blocking Wnt signaling prevents WIHN, while enhancing Wnt signaling in the epidermis sharply increases the number of regenerated follicles after wounding (*7*). Recently, Harn *et al.* demonstrated that optimal tissue rigidity determines where WIHN occurs within the healed wound (*4*). During scab detachment, this permissive zone for hair regeneration in the wound has a tissue rigidity range of Young’s modulus (*E*) = 5 to 15 kPa (*4*).

β-Catenin, the main downstream effector of Wnt signaling, fulfills a unique dual role by serving as a transcription factor and a structural protein (*10*). Adherens junctions (AJs) rich in β-catenin physically link the cytoskeletal actin network of neighboring epithelial cells (*11*). At a tissue scale, AJ-mediated linkages rapidly transmit force between interconnected cells to establish long-range mechanical coupling—the key mechanism behind coordination of force-dependent behaviors within large multicellular clusters (*12*, *13*). Under intense mechanical load, such as those seen during bone formation and tumorigenesis, β-catenin translocates to the nucleus in a force-dependent manner (*14*, *15*). Outside such extreme environments, Wnt signaling’s mechanosensitive function remains elusive for most biologically relevant conditions. Whether Wnt signaling performs a mechanoregulatory role that contributes to regeneration has not been elucidated.

In this study, we interrogated the relationship between mechanotransduction and canonical Wnt signaling using the murine WIHN model of regeneration. Since Wnt activation precedes the emergence of optimal tissue rigidity during WIHN, we investigated whether Wnt signaling affects mechanoregulation of regeneration by characterizing tissue mechanics in wild-type (WT) and genetically modified mice with amplified Wnt signaling. As expected, neogenic hair follicles were only observed within a small central zone of the wound in WT mice, while Wnt-enhanced mice regenerated hundreds of hair follicles over a large area—encompassing nearly half the total wound size in most cases. Atomic force microscopy (AFM) revealed that the tissue rigidity of wounds in Wnt-enhanced mice was within the optimal range for WIHN. On the basis of these findings, we hypothesized that Wnt signaling attenuates mechanotransduction and the substrate rigidity response during wound healing. To test this idea, we manipulated Wnt signaling and assessed its impact on mechanosensitive cell behavior and biomechanical tissue properties. Wnt signaling suppressed the rigidity response at cellular and tissue levels by orchestrating large-scale reorganization of cytoskeletal actin and augmented mechanical cooperativity by recruiting AJs. Wnt-dependent attenuation of the substrate-rigidity response was present in epidermal keratinocytes but absent in dermal fibroblasts, which lack the robust network of AJs found in epithelial cells (*16*, *17*). Collectively, our findings unveil a previously undiscovered mechanoregulatory function of Wnt signaling that promotes regeneration.

## RESULTS

### Wnt signal amplification promotes WIHN by optimizing tissue rigidity

**Fig. 1.**
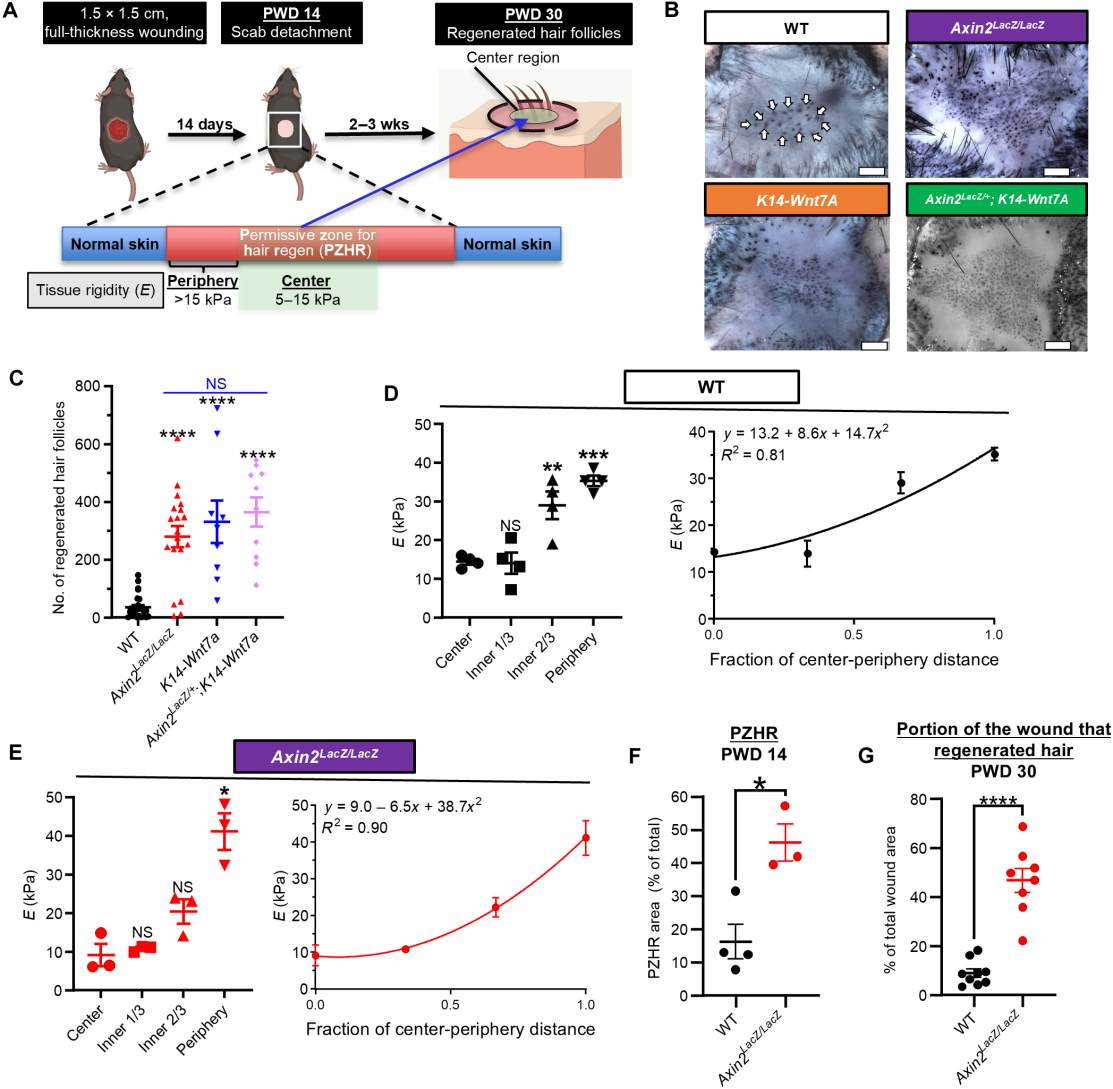
Amplified Wnt signaling optimizes tissue rigidity to promote hair regeneration. (**A**) WIHN model, characterized by hair follicle regeneration in full-thickness wounds. During scab detachment, a PZHR with optimal tissue rigidity for WIHN emerges in the wound’s center region. Subsequent hair placode formation occurs exclusively within the PZHR. wks, weeks. (**B**) WIHN in mice with amplified Wnt signaling. Representative images of *Axin2^LacZ/LacZ^*, *K14-Wnt7a*, *Axin2^LacZ/+^; K14-Wnt7A*, and WT control mouse skin stained with AP to detect hair follicle dermal papilla. Examples of regenerated hair follicles (arrows) in WT control. Scale bars, 0.6 mm. (**C**) Quantification of neogenic hair follicles in healed wounds of mice with amplified Wnt signaling and WT mice. *n* = 9 to 25 mice per group. NS, not significant. (**D** and **E**) Tissue rigidity is attenuated in Wnt-amplified wounds. Spatial tissue mechanics were assessed using AFM on PWD 14 (left), and *E* values were calculated to perform quadratic regression analysis (right) from (D) WT control and (E) *Axin2^LacZ/LacZ^* mice. For spatial tissue mechanics, *E* at each location of the wound was compared to that of the wound center (fraction of center-periphery distance, 0). Regression equation and *R*^2^ values are shown. *n* = 3 to 4 mice per group. (**F** and **G**) Amplified Wnt signaling enlarges the wound area with optimal *E* for WIHN. Compared to WT control, *Axin2^LacZ/LacZ^* mice displayed (F) a larger PZHR area on PWD 14 (*n* = 3 to 4 mice per group) and (G) higher percentage of the wound containing regenerated hair follicles, calculated by dividing the area containing regenerated hair follicles by the total area of the healed wound on PWD 30 (*n* = 8 to 9 mice per group). Increased PZHR area on PWD 14 was predictive of enhanced WIHN observed on PWD 30. Data represented as means ± SEM. *P < 0.05, **P < 0.01, ***P < 0.001, and ****P < 0.0001.

A permissive zone for hair regeneration (PZHR, Fig. 1A), comprising the centermost portion of the wound with ideal rigidity for WIHN [*E* = 5 to 15 kPa; (*4*)], emerges during scab detachment and determines the future site of regeneration. Since Wnt activation at least 2 days before scab detachment is indispensable for hair placode formation during WIHN (*7*, *8*), we hypothesized that Wnt signaling directly affects biomechanical properties of wounds that regulate regeneration.

To test this, we induced WIHN on transgenic mice with amplified Wnt signaling and characterized their tissue mechanics using AFM on the day of scab detachment, which occurs around postwound day (PWD) 14. Signal amplification was attained through epidermal Wnt ligand overexpression [*K14-Wnt7a*; (*7*)] or the loss of Axin2, a downstream Wnt target that acts as a negative regulator of the Wnt signaling pathway (*18*) (*Axin2^LacZ/LacZ^*). C57BL/6J (B6, WT) mice were used as controls.

Both *K14-Wnt7a* and *Axin2^LacZ/LacZ^* mice demonstrated robust WIHN with hundreds of regenerated hair follicles. The number of regenerated hair follicles in *Axin2^LacZ/LacZ^*, *K14-Wnt7a*, and *Axin2^LacZ/+^; K14-Wnt7a* was ~10-fold that of WT mice (Fig. 1, B and C). Regenerative capabilities were similar between these two genetic models. Regenerated hair follicles in WT mice were confined to a small center region of the wound, but this regenerative region (i.e., PZHR) was greatly enlarged in Wnt-enhanced mice. In *Axin2^LacZ/LacZ^* mice, the density of regenerated hair follicles was also elevated (fig. S1A).

Heterozygotes of the *Axin2^LacZ^* strain (*Axin2^LacZ/+^*) are widely used as Wnt reporter mice (*8*, *18*, *19*). Although these mice are designed to faithfully reflect the endogenous level of Wnt signaling (*18*), we identified an unexpected increase in their capacity for WIHN, which was nearly tripled because of enhanced Wnt signaling (fig. S1, B and C). We were unable to find prior literature discussing amplified Wnt activity in this Wnt reporter. Validating our findings in other organ systems will be valuable to potentially rule out an organ-specific effect.

Since wounds in Wnt-enhanced mice displayed WIHN over a larger area than the controls, we tested whether Wnt signaling expands the PZHR in *Axin2^LacZ/LacZ^* mice. To calculate the PZHR area as a fraction of total wound area, AFM measurements were obtained between the wound center and the periphery using skin samples (i.e., epidermis and dermis) harvested from *Axin2^LacZ/LacZ^* and WT mice during scab detachment (fig. S2, A and B). AFM-derived *E* values (fig. S3) were fitted to a quadratic regression function (*R*^2^ = 0.8 to 0.9, Fig. 1, D and E).

The PZHR area in *Axin2^LacZ/LacZ^* mice was nearly triple that of WT (Fig. 1F). The difference in PZHR area on PWD 14 was consistent with the elevated neogenic hair follicle count and the enlarged portion of the wound that regenerated hair follicles seen on PWD 30 (Fig. 1G). The difference in PZHR area for WT and *Axin2^LacZ/LacZ^* mice was not due to an intrinsic difference in their skin rigidity, which was indistinguishable based on AFM measurements of their normal skin (fig. S2c).

Overall, these findings suggest that Wnt signaling optimizes wound rigidity to promote WIHN.

### Wnt attenuates substrate rigidity response and mechanotransduction in the epidermis

To dissect the mechanism underlying Wnt-driven mechanoregulation of regeneration, we interrogated the molecular machinery of mechanotransduction. The fundamental cornerstone of mechanosensitivity is the substrate rigidity response, characterized by the proportional relationship between mechanotransduction and substrate rigidity (*5*). On stiffer substrates, increased mechanotransduction promotes actomyosin recruitment to elevate cell rigidity (fig. S2D).

Since Wnt signaling expanded the PZHR area, we tested whether Wnt signaling suppresses the substrate rigidity response to soften the tissue. To test whether the observed difference in tissue rigidity arose from the epidermis or the dermis, we isolated murine epidermal keratinocytes and dermal fibroblasts and then plated them on biocompatible silicone dishes with tunable substrate rigidity. By varying the degree of silicone cross-linking, substrate *E* was tuned to match the rigidity of the wound center (8 kPa) and periphery (32 kPa). Cells were treated with CHIR99021 (CHIR), a Wnt agonist that inhibits glycogen synthase kinase 3 (GSK-3) (*20*), and AFM was carried out.

**Fig. 2.**
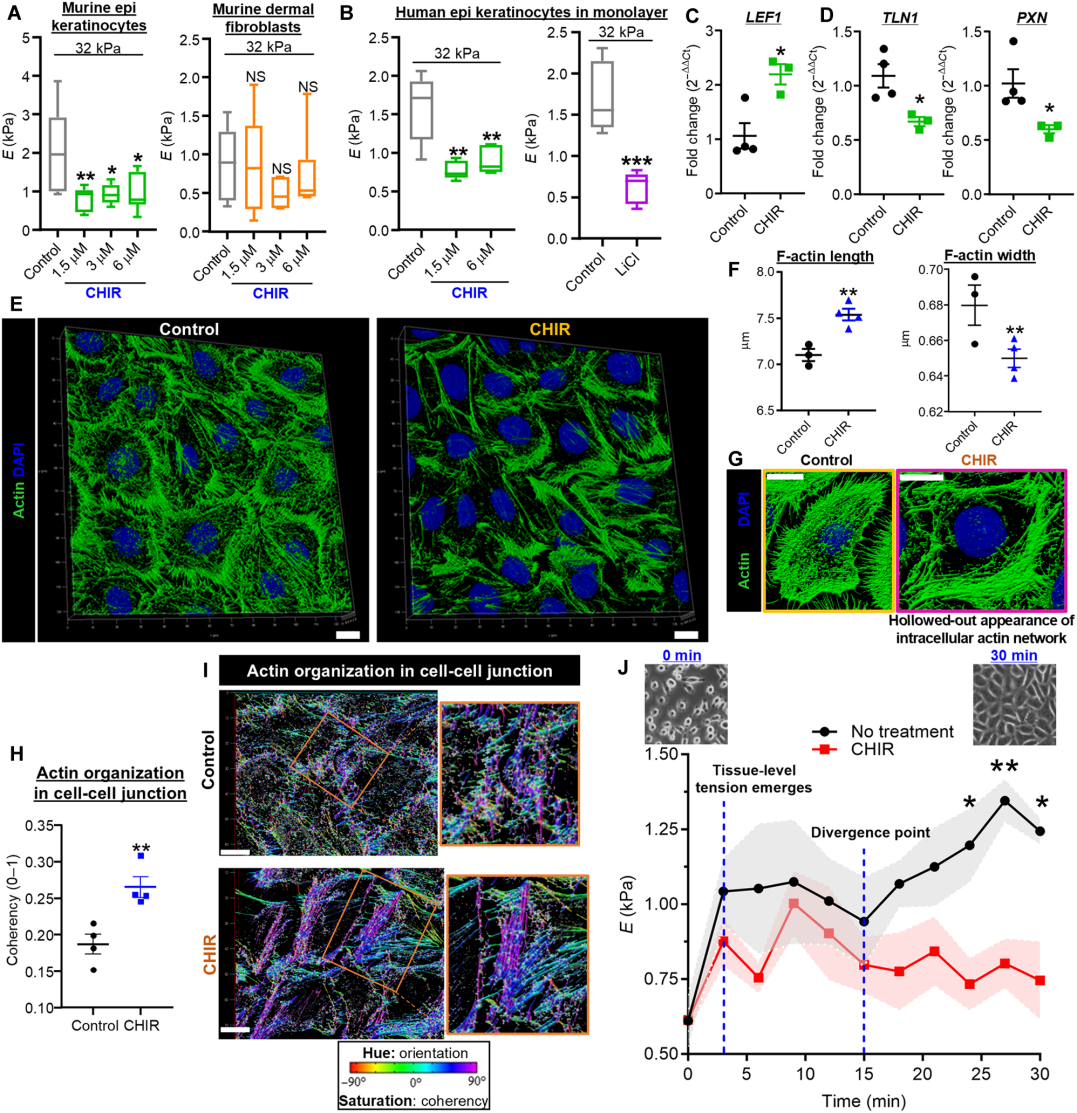
Wnt attenuates mechanotransduction and tissue-level tension in keratinocytes through large-scale reorganization of cytoskeletal actin. (**A**) Wnt-mediated attenuation in substrate rigidity response in murine epidermal keratinocytes but not dermal fibroblasts. Cells grown to 20 to 30% confluence. Substrate *E* = 32 kPa. *n* = 5 to 7 force maps per condition. (**B**) Attenuated substrate rigidity response in human keratinocytes in a monolayer after treatment with CHIR or LiCl. Substrate *E* = 32 kPa. *n* = 5 to 6 force maps per condition. (**C** and **D**) Wnt activity and mechanotransduction in CHIR-treated human keratinocytes. qPCR for (C) *LEF1* and (D) *TLN1* and *PXN*. *n* = 3 to 4 dishes per group. (**E** to **G**) Wnt orchestrates large-scale actin reorganization. (E) Representative 3D actin network for human keratinocytes in a monolayer with or without CHIR treatment. Scale bars, 10 μm. (F) Length and width of actin filaments. *n* = 3 to 4 dishes (one field per dish, 900 to 1200 actin filaments from 13 to 23 cells per field). (G) Representative phalloidin staining with intracellular actin architecture rendering a hollowed-out distribution—sparse in the center and rich in cell-cell junctions at the periphery—in CHIR-treated keratinocytes. Scale bars, 10 μm. (**H** and **I**) Altered junctional actin organization in CHIR-treated cells. (H) Coherency (1, perfect alignment; 0, isotropic conditions) of junctional actin filaments. *n* = 4 dishes per group. (I) Representative phalloidin staining in hue-saturation-brightness (HSB) mode. HSB: hue, orientation; saturation, coherency; brightness from the source image. Scale bars, 10 μm. (**J**) Time-resolved AFM. Monolayer dissociation without cell detachment (top left) after transient calcium deprivation. Calcium and CHIR reintroduced at *t* = 0 min, followed by serial AFM. Monolayer restoration, 30 min after calcium repletion, in both groups (top right). Tissue-level tension emerges in control, not in the CHIR group. *n* = force maps obtained serially in four locations per group. Data represented as means ± SEM. Box-and-whisker plots: central line, median; box, interquartile range [25th (Q1) to 75th (Q3) percentile]; whiskers, 10th and 90th percentiles. *P < 0.05, **P < 0.01, ***P < 0.001, and ****P < 0.0001.

Notably, the substrate rigidity response was highly attenuated in CHIR-treated epidermal keratinocytes but not dermal fibroblasts (Fig. 2A). An appropriate substrate rigidity response was seen in untreated controls (fig. S4, A and B). Using normal human epidermal keratinocytes (NHEKs) grown in a monolayer (fig. S2E), we confirmed our finding with two different GSK-3 inhibitors [CHIR and LiCl; (*21*)] (Fig. 2B). While treating keratinocytes with a Wnt agonist lowered their rigidity, treating them with a Wnt antagonist, DKK4 [Dickkopf-related protein 4; it functions as a negative feedback inhibitor for Wnt signaling during hair placode formation; (*22*)], did not result in cell stiffening (fig. S4F). This finding suggests that suppressing Wnt signaling in the absence of Wnt activators (i.e., “Wnt-off state”) does not affect the rigidity response. Wnt-induced inhibition of cellular mechanotransduction in CHIR-treated keratinocytes was confirmed using quantitative polymerase chain reaction (qPCR) for genes encoding members (*TLN1* and *PXN*) of the focal adhesion complex, the central hub of mechanotransduction (*23*) (Fig. 2, C and D).

Since the substrate rigidity response was unaffected by Wnt signaling in dermal fibroblasts, we sought to validate this finding in vivo using WIHN. To rule out dermal contribution to the altered tissue mechanics seen in *Axin2^LacZ/LacZ^* mice, we probed for ECM alterations using second harmonic generation (SHG) microscopy (*24*). No significant differences were identified between WT and *Axin2^LacZ/LacZ^* mice in their collagen network organization or collagen fiber properties (table S1). Although the wound’s center portion was less rigid than the periphery, collagen density was relatively uniform throughout the wound (fig. S1D).

ECM’s bulk mechanical properties, including the Young’s modulus, are primarily driven by collagen—the most abundant component of the dermal ECM (*25*, *26*). While the collagen network revealed no signs of altered stiffness in the dermal microenvironment on PWD 14, we also considered the role of elastin—the other major fibrous component of the ECM (*25*). However, Luna staining (fig. S1E) of healed wounds in both WT and *Axin2^LacZ/LacZ^* mice revealed little to no elastin fibers—which are not observed until at least PWD 20 to 30 in full-thickness wounds (*27*).

These findings demonstrate that Wnt signaling inhibits mechanotransduction to reduce the rigidity response in the epidermis without inducing significant dermal changes.

### Wnt-driven actin reorganization lowers tissue-level tension in the epidermis

To evaluate the mechanism for the attenuated substrate rigidity response, we assessed the three-dimensional (3D) architecture of actin—the core element of mechanotransduction that governs cellular rigidity (*28*). Increased Wnt signaling in keratinocytes resulted in a profound reorganization of cytoskeletal actin (Fig. 2, E to I). Intracellular actin distribution became sparse in the center and concentrated and organized in the periphery at cell-cell junctions to render a hollowed-out appearance. Longer and thinner actin filaments were seen in CHIR-treated keratinocytes—suggesting reduced actomyosin contraction that was consistent with their attenuated substrate rigidity response.

To confirm these findings, we stained for phosphorylated myosin light chain 2 (MLC2) at its serine 19 residue, which promotes actomyosin contractility (*29*, *30*). While phosphorylated MLC2 (phospho-MLC2) is abundantly expressed at force-generating regions of epithelial cells (e.g., cleavage furrows in cells undergoing mitosis and contractile “purse string” of actomyosin filaments that form during the closure of small wounds), it is normally expressed at low levels at cell-cell junctions (*30*). In CHIR-treated keratinocytes, phospho-MLC2 levels were increased in cell-cell junctions and decreased in the cytoplasm (fig. S5)—consistent with the hollowed-out appearance seen in the actin network.

The integrated density of phalloidin staining was comparable between CHIR and control groups (fig. S4C), indicating that the altered actin architecture arose from reorganization with no change in the total amount of actin. Since the intracellular supply of actin is thought to be finite (*31*) with different cellular processes competing for this limited common pool, Wnt signaling likely increased the allocation of actin to cell-cell junctions, which diminished the actin concentration in other parts of the cell.

During monolayer formation of epithelial cells, actomyosin networks of adjacent cells connect and contract to generate tissue-level tension—the main component of the monolayer’s rigidity response (*12*). To test the effect of Wnt-mediated cytoskeletal rearrangement on tissue-level tension, we performed time-resolved AFM during monolayer formation after a transient disruption in intercellular adhesions induced by a brief period of calcium deprivation. Human keratinocytes were grown in a monolayer, treated with CHIR, and then incubated in calcium-free media. The remaining calcium was chelated using EDTA to eliminate AJs and tissue-level tension, which led to monolayer dissociation. Immediately after calcium repletion, serial force measurements were obtained as the monolayer reformed. In the untreated control group, tissue-level tension rapidly emerged and became the main contributor to *E* within minutes (Fig. 2J). However, CHIR-treated cells did not exhibit significant tissue-level tension even when the monolayer was visibly reformed.

To determine whether Wnt signaling drives actin reorganization in vivo, we used a fluorogenic, live-cell probe against F-actin on healed wounds harvested as fresh tissue from WT and *Axin2^LacZ/LacZ^* mice on PWD 14. To characterize cytoskeletal changes associated with lower tissue rigidity from the wound epidermis, the fluorescence signal for actin (fig. S6) was measured at the wound’s center region—as well as near the periphery (i.e., between the inner two-thirds and the wound edge). Compared to WT mice, *Axin2^LacZ/LacZ^* mice showed significantly lower levels of actin staining near the wound periphery (fig. S6C). This finding is consistent with our AFM results from the same time point, which demonstrated lower tissue rigidity at the inner two-thirds of the wound with enlarged PZHR area in *Axin2^LacZ/LacZ^* mice. Consistent with our in vitro findings in CHIR-treated keratinocytes (Fig. 2G), the actin architecture from the wound epidermis in *Axin2^LacZ/LacZ^* mice appeared hollowed-out (fig. S6, A and B).

These findings suggest that Wnt signaling triggers actin reorganization to suppress the rigidity response at the tissue scale in the epidermis.

### Wnt reduces nuclear rigidity and shifts intracellular forces toward AJs

**Fig. 3.**
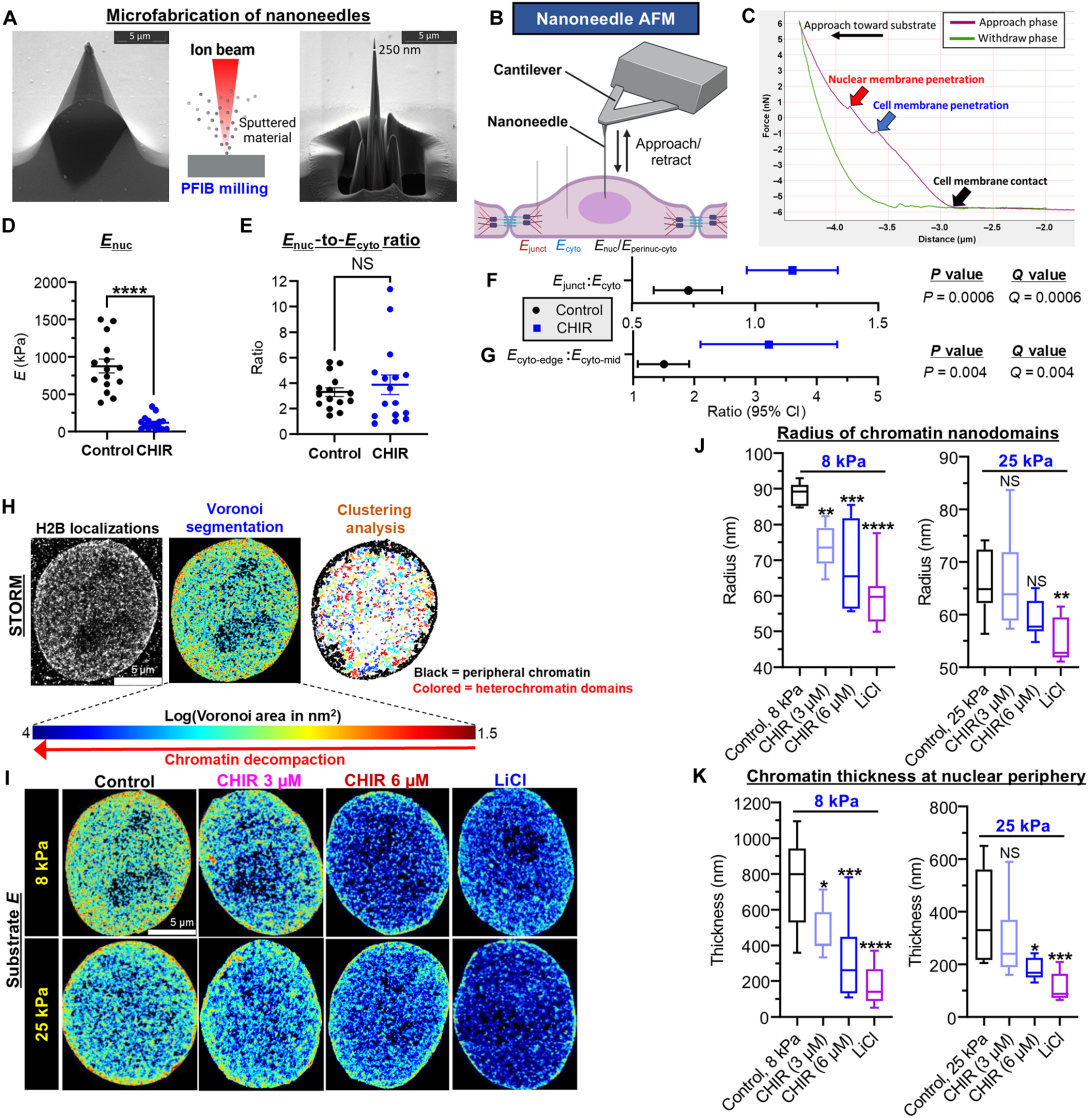
Wnt-driven structural reorganization reduces nuclear rigidity and triggers a shift in intracellular forces toward cell-cell junctions. (**A** to **G**) Nanoneedle AFM. (A) Nanoneedle microfabrication using dual-beam plasma focused ion beam milling. Scanning electron microscopy images before and after milling. Scale bars, 5 μm. (B) Measurement sites. (C) Representative force-distance plot from nanoneedle AFM of the nucleus. Two successive force peaks at the approach phase of the curve indicate successful penetrations of the cell and nuclear membranes. [(D) and (E)] Wnt-dependent chromatin remodeling reduces nuclear rigidity while preserving nucleocytoskeletal mechanical coupling. (D) *E*_nuc_ and (E) the *E*_nuc_:*E*_cyto_ ratio in control and CHIR-treated cells. *n* = 15 to 16 cells per group. [(F) and (G)] Wnt-induced shift in force distribution. Relative increase in rigidity at (F) cell-cell junctions and (G) the monolayer edge, where cell-ECM forces became concentrated. *n* = 15 to 17 cells per group. (**H** to **K**) Wnt-induced chromatin remodeling in human keratinocytes on substrate *E* of normal skin or scar (5 or 25 kPa). (H) STORM analysis of nuclear H2B. Representative H2B localizations (left). Histone nanodomains identified via Voronoi tessellation (middle) and characterized using clustering analysis (right). (I) Representative spatial distribution of histone nanodomains after CHIR/LiCl treatment. In untreated cells, chromatin nanodomains were compact and concentrated at the nuclear periphery—consistent with a state of transcriptional repression. (J) Radius of chromatin nanodomains and (K) chromatin thickness at the nuclear periphery. At normal skin *E*, both concentrations of CHIR and LiCl promoted chromatin architecture conducive to transcription (chromatin decompaction, decreased localization at the nuclear periphery), while on scar-level *E*, the magnitude of Wnt-induced chromatin remodeling was smaller. *n* = 8 nuclei per condition (at least four biological replicates—or four wells—imaged for analysis per condition). Data represented as means ± SEM [(D) and (E)], mean ± 95% CI [(F) and (G)], or box-and-whisker plots. *P < 0.05, **P < 0.01, ***P < 0.001, and ****P < 0.0001.

Since Wnt signaling altered the cellular architecture to affect mechanosensitivity, we measured how this change influences the intracellular distribution of force using nanoneedle AFM—a technique that enables direct intracellular probing of live cells with minimal damage (*32*, *33*) (Fig. 3, A to C). Nanoneedle measurements were obtained from the nucleus (*E*_nuc_), cytoplasm (*E*_cyto_), and cell-cell junctions (*E*_junct_). Successful membrane penetration was confirmed on the force-distance plot using a previously validated approach (*32*–*34*) (see the “Nanoneedle AFM” section in Materials and Methods and fig. S7).

The nucleus, the stiffest organelle, relies on chromatin architecture to rapidly alter its stiffness during periods of mechanical stress (*35*). Prior AFM studies report ~40% reduction in nuclear stiffness after chromatin decondensation induced by broad-spectrum histone deacetylase inhibitors, such as trichostatin A and valproic acid (*36*–*38*). However, this finding was based on indirect measurements from the cell membrane overlying the nucleus or from extracted nuclei, which lack vital nucleocytoskeletal connections that regulate the nuclear response to mechanical stress (*39*). Furthermore, it is largely unknown how chromatin decondensation at more selective regions of the genome (e.g., Wnt target genes) affects nuclear mechanics.

Our nanoneedle AFM findings revealed that Wnt agonist treatment (CHIR, 6 μM) triggers a nearly 10-fold drop in *E*_nuc_ (Fig. 3D). Although CHIR-treated cells were less rigid overall (fig. S7C), the ratio between *E*_nuc_ and *E*_cyto_ remained constant (Fig. 3E)—suggesting intact nucleocytoskeletal mechanical coupling (*39*). Increased organization and recruitment of junctional actin filaments elevated the ratio between *E*_junct_ and *E*_cyto_, indicating a shift in intracellular forces toward cell-cell junctions (Fig. 3F).

These data suggest that Wnt-induced changes in cellular architecture increase the relative rigidity of cell-cell junctions and reduce nuclear rigidity. By using a nanoneedle as a probe, we were able to obtain force measurements with minimal perturbation to the native architecture of the cell (e.g., preserved nucleocytoskeletal connections, which are lacking in extracted nuclei from lysed cells). Since Wnt signaling attenuates cytoplasmic rigidity (fig. S7C), the preserved nucleocytoskeletal connections—which enable force transmission between the nucleus and the cytoplasm—likely explain why the magnitude of nuclear softening was greater than what was previously reported in extracted nuclei using histone deacetylase inhibitors.

### Wnt-induced chromatin remodeling is blunted in a scar-like environment

Our nanoneedle AFM results indicated that Wnt-induced chromatin decondensation softens the nucleus for a given substrate rigidity. On different substrate rigidities, it was recently demonstrated via super-resolution microscopy [stochastic optical reconstruction microscopy (STORM)] that cells display signs of transcriptional repression (more compact chromatin nanodomains and increased chromatin thickness at the nuclear periphery, a transcriptionally repressive area of the nucleus) on softer substrates (*40*). However, the significance of this finding—especially in the context of regeneration—is unclear.

Nuclear β-catenin induces chromatin remodeling at Wnt target loci, which leads to transcriptional activation of Wnt target genes (*41*, *42*) that play a crucial role during regeneration (*10*). To test the effect of substrate rigidity on Wnt-induced chromatin remodeling, we grew human keratinocytes on substrate rigidities of normal skin (8 kPa) and scar (25 kPa) and used STORM to detect histone H2B expression after treatment with CHIR (3 and 6 μM) or LiCl (Fig. 3, H to K; and fig. S4, D and E).

On normal skin rigidity, LiCl and both concentrations of CHIR promoted chromatin architecture conducive to transcription (less compact chromatin nanodomains and decreased chromatin thickness at the nuclear periphery, Fig. 3, J and K). LiCl was more effective at inducing chromatin remodeling than CHIR. On scar *E*, however, the Wnt-induced changes in chromatin architecture were far more subdued, and no architectural changes were seen with the lower concentration of CHIR.

These findings suggest that Wnt-induced chromatin remodeling is blunted in a scar-like environment. This may mask the more subtle effects on chromatin architecture induced by low levels of Wnt activators during early regeneration [e.g., Wnt2a ligand produced from the wound dermis during early WIHN; (*8*)], which would contribute to the lack of regeneration seen on a stiff tissue environment.

### Wnt signaling promotes AJ formation

GSK-3 inhibition leads to cytoplasmic accumulation of β-catenin, which translocates to the nucleus (*21*) (fig. S4F), but it is uncertain whether significant crossover also enriches the membrane reserve of β-catenin to heighten AJ formation (*43*, *44*). Since AJs are primary anchoring sites of actin filaments at cell-cell junctions (*28*), we reasoned that Wnt-induced local enrichment of junctional actin was supported by increased AJ formation.

**Fig. 4.**
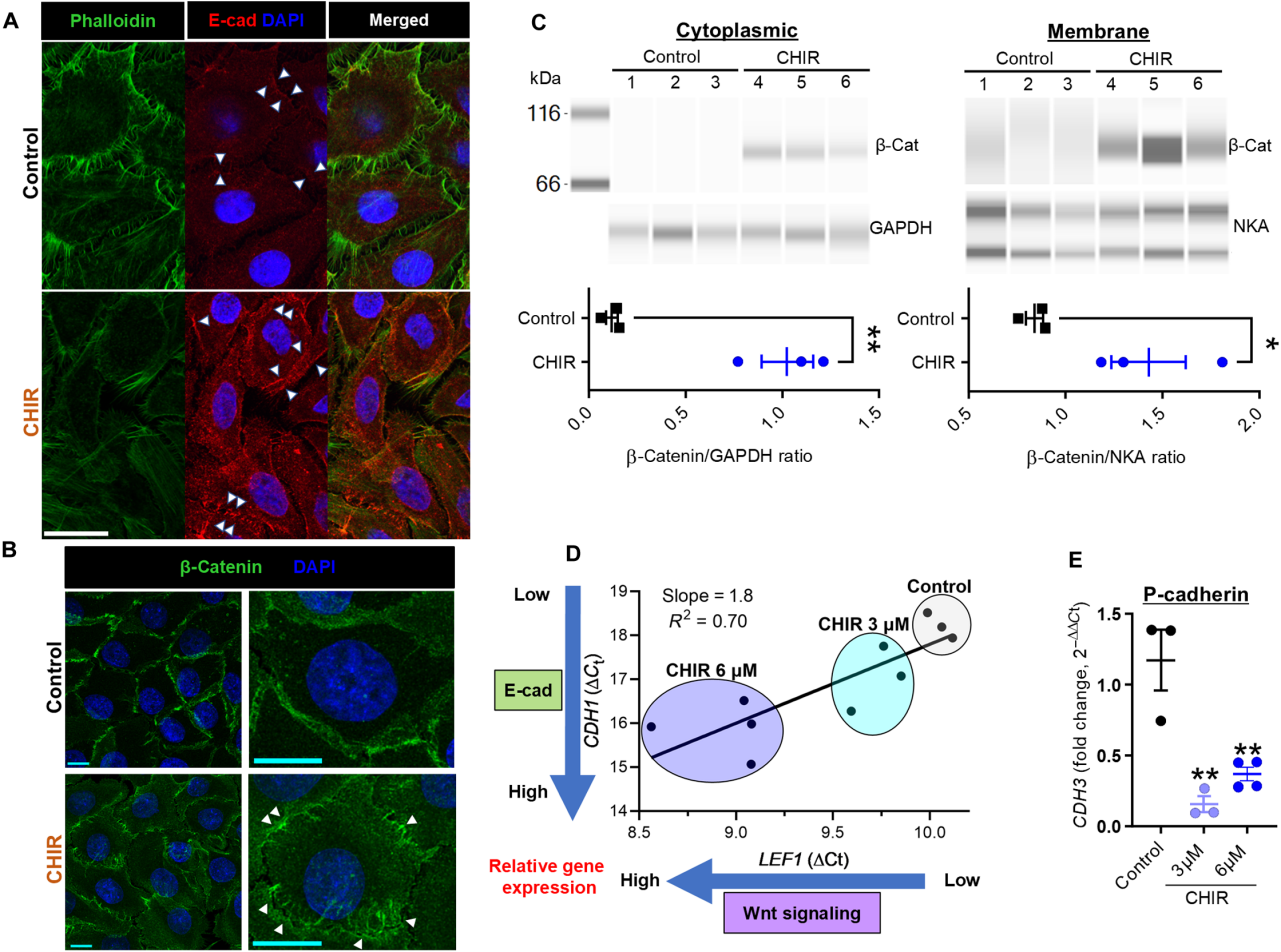
Canonical Wnt signaling up-regulates AJ recruitment in epidermal keratinocytes. (**A**) Representative images of phalloidin (green) and E-cadherin (red) staining in CHIR-treated human keratinocytes. Increased expression of membrane E-cadherin (white arrowheads) after CHIR treatment. Scale bars, 20 μm. (**B**) Representative images of β-catenin (green) staining in CHIR-treated human keratinocytes. Increased membrane β-catenin (arrowheads) after CHIR treatment. Scale bars, 20 μm. (**C**) Capillary electrophoresis immunoblots for β-catenin expression in cytoplasmic and membrane fractions of CHIR-treated human keratinocytes. Cytoplasmic loading control, GAPDH. Membrane loading control, Na/K-ATPase (NKA). Two bands for NKA correspond to intact form (150 kDa) and α1 subunit (100 kDa) of NKA (*95*), and the ratio reflects the sum of both bands. After CHIR treatment, membrane β-catenin expression was increased. (**D** and **E**) qPCR assessment for *CDH1* and *CDH3* of CHIR-treated human keratinocytes. (D) Linear regression analysis revealed a strong, linear relationship between *CDH1* and *LEF1* expression. *LEF1* induced by CHIR in a dose-dependent manner, with (E) a corresponding decrease in *CDH3* in the same set of samples. *n* = 3 to 4 dishes per group. Data represented as means ± SEM. *P < 0.05 and **P < 0.01. Unprocessed blots are provided.

Confocal microscopy of CHIR-treated human keratinocytes (Fig. 4, A and B) revealed increased membrane expression of AJ components β-catenin and E-cadherin [dominant cadherin isoform in the interfollicular epidermis; (*45*)]. Increased β-catenin in the plasma membrane was confirmed using capillary electrophoresis (Simple Western) after subcellular fractionation (Fig. 4C).

Nascent AJs form as discrete puncta (i.e., punctate AJs) along the cell membrane (*46*–*48*). In addition to E-cadherin, these punctate AJs recruit actin regulatory proteins [e.g., vinculin and vasodilator-stimulated phosphoprotein (VASP)] to form the “adhesion zipper”—rows of punctate AJs from opposing cell membranes that interlock together to form a mature AJ with linear morphology (i.e., linear AJ) (*48*).

To assess whether Wnt signaling induces the formation of nascent AJs, we stained for E-cadherin, vinculin, and VASP in human keratinocytes treated with CHIR (6 μM). The cells were plated at ~30% confluency, incubated overnight, and treated with CHIR for 1, 3, 6, or 24 hours before fixation and staining. Confocal microscopy revealed increased formation of adhesion zippers in keratinocytes after 1 to 3 hours of treatment with CHIR (fig. S8). Vinculin expression largely mirrored that of its binding partner, VASP (*49*), and both were found at sites of adhesion zipper formation (fig. S8A). After 6 hours of treatment, adhesion zippers began to merge together and form linear AJs (fig. S8B).

After 48 hours of treatment, E-cadherin expression on the cell membrane was measured by analyzing the line profile of fluorescence intensity—which revealed a significant increase in membrane E-cadherin expression in the CHIR-treated group (fig. S4, G and H).

Since β-catenin binds the cytoplasmic domain of E-cadherin to form AJs (*50*), we sought to confirm the joint increase in E-cadherin and β-catenin by measuring the Förster resonance energy transfer (FRET) interaction. By using fluorescence lifetime imaging microscopy (FLIM) to determine the FRET interaction between E-cadherin and β-catenin, we performed FLIM-FRET—a powerful technique for precisely quantifying protein-protein interactions in cells (*51*, *52*). Since FRET efficiency increases with lower intermolecular distance, stronger protein-protein interactions, and favorable conformational changes, an increase in FRET efficiency is a strong indicator of binding events between two proteins (*51*, *52*).

FLIM-FRET studies were carried out in human keratinocytes after 48 hours of treatment with CHIR. By using fluorescently labeled antibodies targeting the cytoplasmic domain of E-cadherin versus the 100 C-terminal residues of β-catenin [i.e., portion of the protein involved in forming the interaction surface between E-cadherin and β-catenin (*50*)], β-catenin and E-cadherin were fluorescently labeled as the FRET donor (Alexa Fluor 488) and acceptor (Alexa Fluor 555), respectively [see the schematic of experimental design in fig. S9 (A and B)]. Since CHIR triggered the formation of adhesion zippers that matured into linear AJs, we hypothesized that FRET efficiency between β-catenin and E-cadherin will be higher in the CHIR-treated group. FRET efficiency measurements from cell-cell junctions revealed a threefold increase in the CHIR-treated group (fig. S9, C and D).

To rule out a global overexpression of cell-cell adhesion proteins, we examined desmoglein expression and found no change (fig. S4I). A Wnt-driven increase in E-cadherin (*CDH1*) was validated further via qPCR, which revealed a strong, linear relationship between *LEF1* and *CDH1* with proper attenuation in *CDH3* [encoding P-cadherin, which exhibits an inverse relationship with E-cadherin in epidermal keratinocytes; (*45*)] (Fig. 4, D and E). To test whether the transcriptional up-regulation of E-cadherin induced by CHIR resulted in translational up-regulation, total cell fluorescence measurements of E-cadherin were compared between CHIR-treated cells and control cells. Total cell fluorescence measurements of E-cadherin were significantly higher in the CHIR-treated group (fig. S4J).

Next, we assessed whether Wnt signaling promotes AJ recruitment in vivo by characterizing the expression pattern of E-cadherin and β-catenin in wounds harvested on PWD 14 from WT and *Axin2^LacZ/LacZ^* mice. The wound epidermis of WT mice demonstrated robust membrane expression of β-catenin in the basal but not the suprabasal layer, which had weaker staining, as previously described (*45*). In stark contrast, wounds from *Axin2^LacZ/LacZ^* mice revealed exuberant membrane expression of β-catenin in both the basal and suprabasal layers (fig. S10, A and B).

While the basal layer expresses both E- and P-cadherin, the suprabasal layer relies on E-cadherin exclusively to maintain its AJs (*45*). In *Axin2^LacZ/LacZ^* mice, E-cadherin expression at intercellular junctions showed a similar pattern of robust increase as that of β-catenin (fig. S10A). In *Axin2^LacZ/LacZ^* mice, the thickness of intercellular junctions expressing E-cadherin (fig. S10C) was significantly elevated—suggesting that increased canonical Wnt signaling during wound healing promotes AJ recruitment in the wound epidermis in vivo. Increased nuclear β-catenin expression was also observed (fig. S10D).

These in vitro and in vivo data provide rigorous evidence that Wnt signaling promotes actin recruitment to cell-cell junctions—leading to increased AJ formation in keratinocytes.

### Wnt-driven AJ recruitment augments collective cell behavior

AJs strengthen intercellular adhesion, the key component of mechanical cooperativity (*11*, *12*, *28*, *53*). Multicellular keratinocyte colonies with strong intercellular adhesions effectively contract as a single unit to concentrate their cell-ECM forces around the colony rim (*54*). In these colonies, increased cell-cell forces exerted at cell-cell contacts are counterbalanced by increased cell-ECM forces [i.e., the magnitude of vector sum of these forces equates to zero (*55*)]. Since Wnt signaling recruited AJs, we hypothesized that Wnt signaling promotes mechanical cooperativity and results in higher cell-ECM forces around the colony rim.

**Fig. 5.**
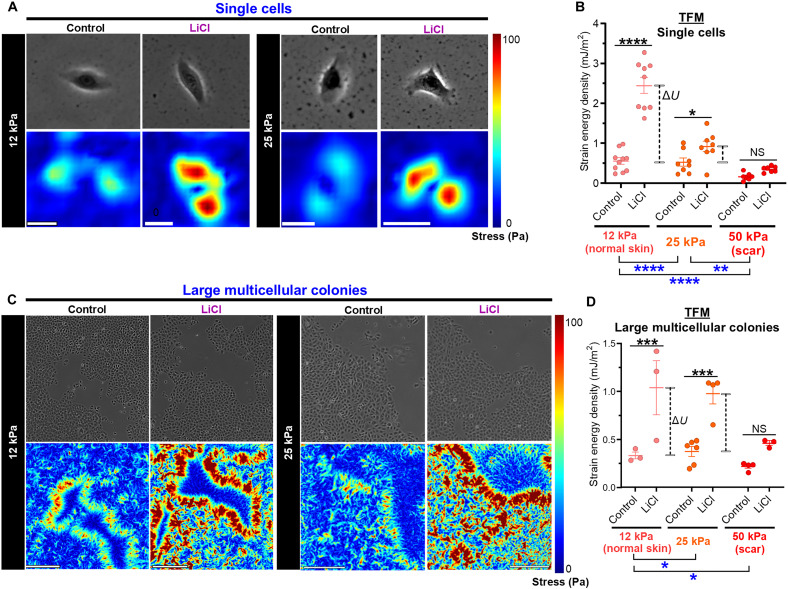
Wnt-driven AJ recruitment augments coordination of cellular forces with the greatest impact on large multicellular colonies grown on soft substrates. (**A** and **B**) TFM of LiCl-treated keratinocytes grown as single cells. (A) Representative traction force stress maps of human keratinocytes cultured at substrate *E* = 12 or 25 kPa. Scale bars, 40 μm. (B) Strain energy density plots of human keratinocytes cultured at substrate *E* = 12, 25, or 50 kPa. Differences in strain energy listed as Δ*U*, which was more pronounced on lower-rigidity substrates with higher susceptibility to cell-induced deformation. *n* = 7 to 10 cells per condition. For each condition, three to four biological replicates were included (three to four wells per group). (**C** and **D**) TFM of LiCl-treated keratinocytes grown as large multicellular colonies. Wnt signaling led to increased concentration of cell-ECM forces at the monolayer’s edge. (C) Representative traction force stress maps of human keratinocytes cultured at substrate *E* = 12 or 25 kPa. Scale bars, 500 μm. (D) Strain energy density plots of human keratinocytes cultured at substrate *E* = 12, 25, or 50 kPa. Differences in strain energy listed as Δ*U*. *n* = 3 to 6 colonies per condition. For each condition, three to four biological replicates were included (three to four wells per group). Data represented as means ± SEM. *P < 0.05, ***P < 0.001, and ****P < 0.0001.

Traction force microscopy (TFM) revealed elevated cell-ECM forces in single cells and multicellular clusters after LiCl treatment, but larger differences were seen in the latter (Fig. 5). Wnt signaling increased traction forces at the monolayer’s edge, which was more pronounced on lower-rigidity substrates with greater susceptibility to cell-induced deformation. We confirmed our finding by comparing rigidity measurements between the middle of the monolayer (*E*_cyto-mid_) and its edge (*E*_cyto-edge_) using nanoneedle AFM. The ratio between *E*_cyto-edge_ and *E*_cyto-mid_ more than doubled after Wnt agonist treatment during nanoneedle AFM (Fig. 3G), which was consistent with increased cell-ECM forces seen around the colony rim during TFM.

**Fig. 6.**
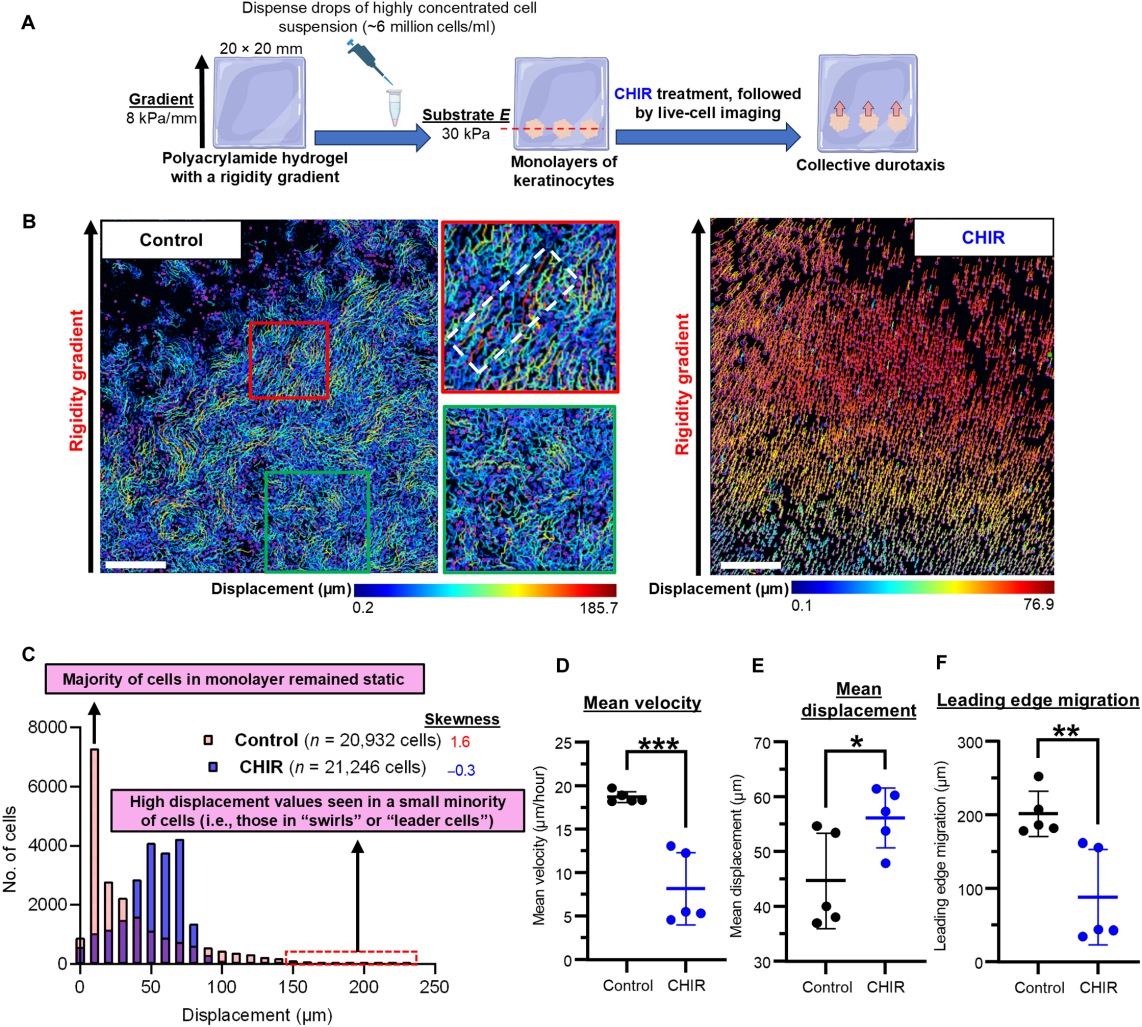
Wnt signaling augments collective durotaxis. (**A**) Characterization of collective durotaxis in human keratinocytes cultured as confluent monolayers on hydrogels with a rigidity gradient (south to north). On locations of known substrate *E* (~30 kPa), small monolayers were generated by dispensing drops of highly concentrated cell suspension. After 24 hours, CHIR was added, followed by live-cell imaging. (**B**) Representative displacement maps. In the control group (left), most cells remained static and significant displacement was only seen in a small minority—those in the leading edge (red box) of the monolayer and those displaying coordinated swirling motions (green box). In contrast, vast majority of CHIR-treated cells (right) underwent significant migration with reduced speed. (**C**) Frequency distribution of displacement values. Each bar (red, control; blue, CHIR) represents the number of cells with listed displacement value. The distribution was highly skewed to the right in the control group (skewness, 1.6) to reflect the small minority of cells that exhibited high displacement values and majority of cells that remained static. In comparison, the distribution was Gaussian in the CHIR-treated group (skewness, −0.3) to reflect the shift in migratory behavior—most cells underwent collective durotaxis at a low speed. (**D** and **E**) Migratory properties of cells. (D) Mean velocity was reduced after CHIR treatment, but (E) mean displacement was elevated since more cells underwent migration. Each point reflects the mean of all cells in a given field (>10,000 cells per field, *n* = 5 separate monolayers per condition). Data from two separate experiments performed under identical conditions. (**F**) Displacement measured from the leading edge of the monolayer. Net migration toward the rigid side of the substrate was seen in both groups, but the displacement of the leading edge was lower in CHIR-treated cells. *n* = 5 separate monolayers per condition. Data represented as means ± SEM. *P < 0.05, **P < 0.01, and ***P < 0.001.

We validated the Wnt-mediated increase in mechanical cooperativity by assessing collective durotaxis—coordinated multicellular migration along a substrate rigidity gradient (*13*, *53*). Collective durotaxis of epithelial cells requires AJs (*13*). Monolayers of human keratinocytes were cultured on hydrogels with a stiffness gradient, and cellular movement was tracked (Fig. 6A).

Collective durotaxis was seen with net movement toward the stiffer side (Fig. 6, B and F). In the control group, large displacements (i.e., total distance traveled by each cell) were only seen in a minority of cells at the leading edge (leader cells) and those displaying coordinated swirling motions (*53*), while the majority (follower cells) remained static. Therefore, the frequency distribution of displacement values was highly skewed to the right (Fig. 6C). In contrast, CHIR-treated cells migrated more slowly, but nearly all cells—both leaders and followers—moved together in unison (Fig. 6, D and E). This shift in migratory behavior was reflected by the Gaussian distribution of displacement values seen in the CHIR-treated group (Fig. 6C). Thus, Wnt signaling increased synchrony during mass migration at the expense of speed.

These findings show that Wnt bolsters mechanical cooperativity to promote collective durotaxis.

## DISCUSSION

Wnt signaling—which has broad biological relevance during development, regeneration, and wound healing (*10*)—is indispensable for hair follicle formation during both embryogenesis (*56*, *57*) and WIHN (*7*, *8*, *58*). Wnt signaling is mediated through β-catenin, which functions not only as a transcription factor but also as a structural component of AJs that coordinate force-dependent cell behaviors (*12*, *53*).

**Fig. 7.**
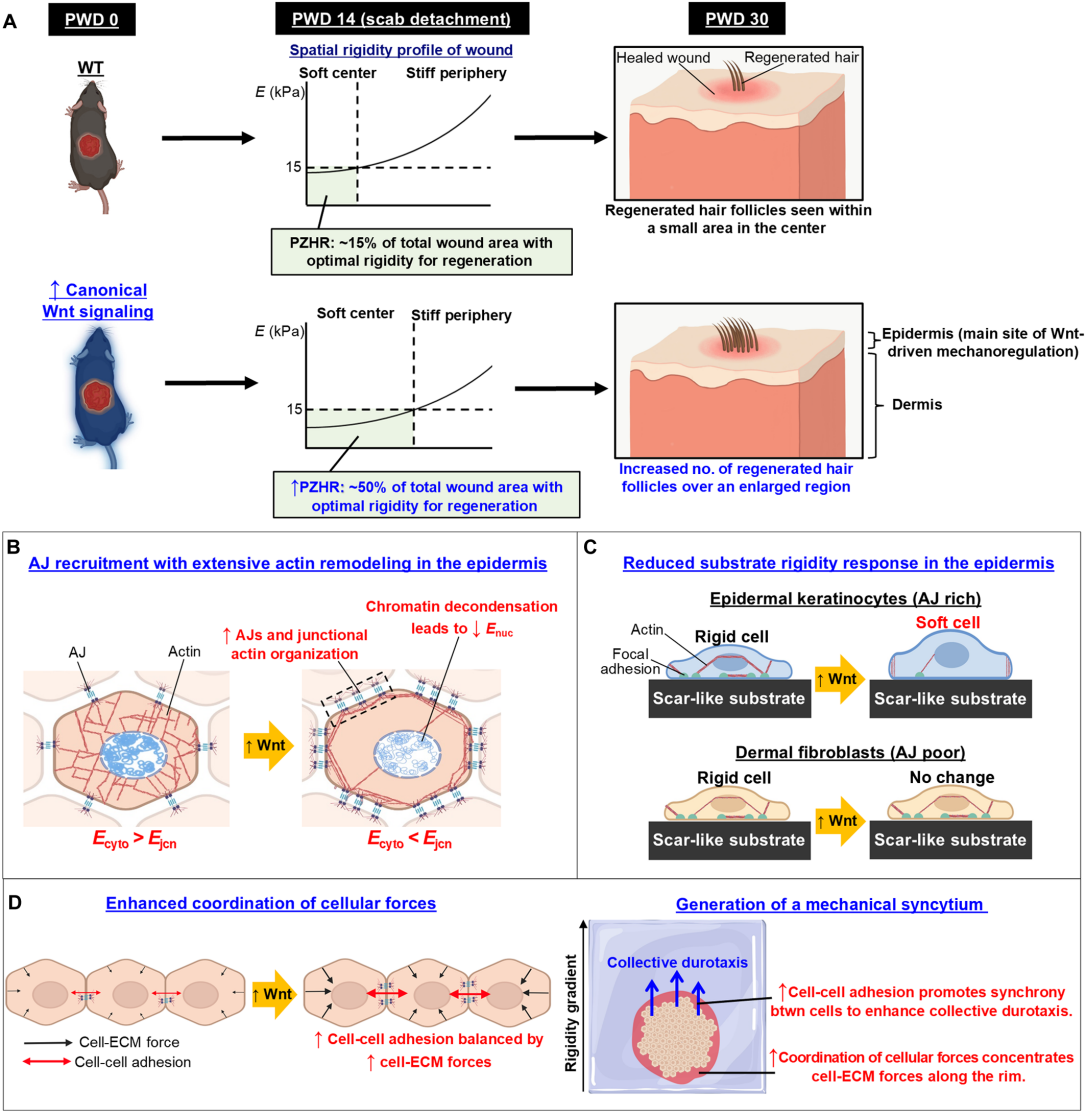
Wnt-induced cadherin-based adhesions generate a mechanical syncytium. (**A**) Wnt signaling optimizes tissue mechanics to amplify the regenerative capacity of a wound. During WIHN, PZHR (area with optimal tissue *E*) emerges in the wound’s center region after re-epithelialization to define the zone capable of hair regeneration. Wnt signaling, which precedes scab detachment by 2 to 3 days, expands the PZHR area severalfold to greatly amplify the regenerative capacity of the wound. (**B** to **D**) Summary of the Wnt-mediated mechanoregulatory axis. (B) Mechanobiological response of keratinocytes to Wnt. β-Catenin serves a dual role as the main downstream effector of Wnt signaling and an essential component of AJs. Increased cytoplasmic β-catenin enhances the formation of AJs, which anchor actin filaments at cell-cell junctions. This triggers a massive shift in intracellular actin architecture to render a “hollowed-out” appearance: sparse in the center and rich in the periphery (i.e., cell-cell junctions), where junctional actin filaments become highly organized. As a result, the entire cell becomes less rigid. However, the drop in rigidity is less pronounced in cell-cell junctions (*E*_jcn_) compared to other cellular compartments (e.g., cytoplasmic rigidity, *E*_cyto_). In the nucleus, Wnt agonist treatment promotes chromatin decondensation, a state that is conducive to transcription of Wnt target genes. This leads to a steep decline in nuclear rigidity (*E*_nuc_). (C) Unlike keratinocytes, fibroblasts lack a robust network of AJs. Thus, Wnt-induced actin remodeling—which is AJ dependent—attenuates mechanotransduction and the substrate rigidity response in epidermal keratinocytes but not dermal fibroblasts. (D) Wnt signaling generates a mechanical syncytium—a cohesive contractile unit with augmented mechanical cooperativity. The Wnt-driven increase in cell-cell adhesion is counterbalanced by higher cell-ECM forces, which becomes concentrated around the rim of multicellular colonies. Increased cadherin-based adhesions, supported by organized junctional actin filaments, enhance collective durotaxis by promoting synchrony between cells over speed.

We uncovered a mechanoregulatory effect of Wnt signaling that provides a mechanistic basis for the previously speculated role of β-catenin as a “molecular bridge” between soluble signaling and mechanosensitivity (*44*). Our findings (Fig. 7) demonstrate that Wnt signaling recruits AJs to alter the actin architecture and suppress the substrate rigidity response in epidermal keratinocytes. At a tissue level, Wnt induced the formation of a mechanical syncytium—a multicellular structure with physical and functional properties of a single collective unit. Collectively, these effects expanded the zone of regeneration within the healed wound and increased the number of neogenic hair follicles.

Our findings suggest that optimal tissue rigidity for WIHN is driven by mechanosensitive changes in the epidermis. Unlike keratinocytes, fibroblasts lack a robust network of AJs (*16*, *17*)—which likely explains their unaltered substrate rigidity response. Despite large differences in tissue rigidity between WT and *Axin2^LacZ/LacZ^* mice, we found no difference in their dermal collagen—the main determinant of the ECM’s bulk mechanical properties (e.g., Young’s modulus) (*25*). Similar findings were previously shown in WT mice on PWD 14, revealing two- to sixfold differences in tissue *E* between the wound center and periphery with minimal differences (~10%) in their dermal collagen properties (*4*). Thus, the tissue findings support the results seen in cells.

Our AFM results and those previously reported by Harn *et al.* (*4*) both showed wounds becoming increasingly more stiff near the wound edge (Fig. 1, D and E). In both studies, the rigidity of the wound edge was severalfold higher than optimal tissue rigidity for WIHN (~5 to 15 kPa). In *Axin2^LacZ/LacZ^* mice, we posit that the attenuated stiffness from Wnt signaling is not potent enough to overcome the high tissue rigidity that emerges at the wound edge (i.e., the effect of Wnt is enough to expand the permissive zone but not enough to overcome the effects of increased stiffness at the wound edge, which is severalfold more rigid than the permissive zone).

Wnt signaling during development and regeneration is often transient and ligand dependent (*7*, *10*). While it was previously demonstrated that epidermal β-catenin down-regulates E-cadherin during hair follicle development in utero, this finding was generated using mice with forced expression of a constitutively active form of β-catenin in all basal epidermal cells (*59*, *60*). These mice form follicular overgrowths and tumors (*59*, *60*) that were not seen in our study. Similarly, the forced expression of activated β-catenin in dermal fibroblasts resulted in exuberant scar formation in wounds (*61*). In contrast, the ligand-dependent models of Wnt amplification (*K14-Wnt7a* and *Axin2^LacZ/LacZ^* mice) used in our studies only increase Wnt signaling in cells capable of responding to Wnt. Thus, constitutive activation models and ligand-dependent models target different cell populations and produce differing results.

Our collective durotaxis and TFM findings demonstrate that Wnt-mediated AJ recruitment enhances mechanical cooperativity and long-range intercellular force transmission. Collective durotaxis, coordinated multicellular migration along a substrate rigidity gradient (*13*), was identified in vivo only recently (*62*). During embryogenesis in *Xenopus laevis*, the neural crest (embryonic stem-cell population) was shown to “chase” a local stiffness gradient formed by the adjacent cranial placode to undergo collective durotaxis (*62*).

WIHN in many ways recapitulates embryogenesis and shares many overlapping features with follicular morphogenesis in utero (*9*, *63*). Hair placode formation [WIHN: PWD 14 (*7*); embryogenesis: ~E14.5 (*63*)] requires actin-dependent force generation (*64*) and involves rearranging/reshaping of existing cells with mitosis playing a limited role (*65*). Through an unknown mechanism, epithelial cells undergo directional migration toward the site of emerging placodes (*64*, *65*). Although Wnt signaling is active during the preplacode stage, it shows uniform dermal expression with no gradient (*66*)—reducing the likelihood of chemotaxis-based guidance at this stage.

Wnt signaling’s effect on collective durotaxis lends valuable mechanistic insight into mechanoregulation of hair follicle formation, which remains poorly understood. Harn *et al.* showed that placode-forming regions were significantly stiffer than their surrounding skin during both embryogenesis (E14) and WIHN (PWD 14) (*4*), but the significance of this finding was uncertain until now. Since the local stiffness gradient between placode-forming regions (stiff) and their surrounding skin (soft) was even more pronounced than the one that triggers durotaxis in *X. laevis* embryos (*4*, *62*), collective durotaxis of epidermal cells may represent one of the earliest drivers of hair follicle formation.

In terms of future directions, using CHIR-treated cells cultured on different substrate rigidities to profile specific changes in gene expression and chromatin accessibility may yield further mechanistic insight into Wnt signaling’s mechanoregulatory function. Since various non-Wnt signaling pathways (e.g., Edar, FGF, and BMP signaling) also become active during hair placode formation (*63*), additional studies are warranted to determine their role in regulating mechanosensitivity. Addressing how these other pathways affect adhesion molecules during and after the cadherin switch [i.e., progressive down-regulation of E-cadherin with up-regulated P-cadherin expression that occurs during hair placode formation; (*67*–*69*)] is also an excellent area for further investigation.

Collectively, our findings demonstrate that Wnt signaling manipulates the molecular machinery of mechanotransduction to generate a conducive environment for regeneration. This knowledge provides a platform for developing therapeutic approaches to treat scarring and other fibrotic disorders. Given Wnt signaling’s indispensable role during regeneration and embryogenesis, we posit that Wnt signaling’s mechanoregulatory function may govern the migration of epithelial cells en masse during these intricate biological processes.

## MATERIALS AND METHODS

### Research animals and WIHN protocol

The protocol for all animal studies underwent review and approval from the Institutional Animal Care and Use Committee (reference no. 803166) at the University of Pennsylvania. The following murine strains with enhanced canonical Wnt signaling were used: (i) *Axin2^LacZ/LacZ^* (RRID: IMSR_JAX:009120), which has a heightened and prolonged Wnt response because of the loss of endogenous negative Wnt regulator Axin2 (*18*), and (ii) *K14-Wnt7a*, a known promotor of WIHN (*7*) with ligand overexpression restricted to the outer root sheath and basal keratinocytes of the interfollicular epidermis.

C57BL/6J (B6) mice were purchased from the Jackson Laboratory. *K14-Wnt7a* mice were retained from a prior study (*7*). The establishment of the *Axin2^LacZ^* colony was aided by a gift of two breeding pairs from M. Ito at New York University. All animals were maintained and housed at the Association for Assessment and Accreditation of Laboratory Animal Care International–accredited animal facility in the Clinical Research Building. The Penn University Laboratory Animal Resources committee supervised the animal conditions. Female and male mice were used in equal ratios.

The WIHN protocol was carried out as previously described (*7*, *8*). The hair on backs of 3-week-old mice was clipped, and 2.25-cm full-thickness wounds were made to the level of panniculus carnosus under isoflurane anesthesia.

### AP staining

For detection of dermal papillae after WIHN, alkaline phosphatase (AP) staining was performed as previously described (*7*) on fresh tissue on PWD 30 after overnight treatment in dispase (2 mg/ml, catalog no. 17105041, Gibco, Waltham, MA) to gently remove the epidermal portion of the healed wound. Images were obtained using either Leica MZFLIII or Leica DFC7000T dissecting scopes, and neogenic hair follicles were counted. WIHN hair density was calculated in 4- to 6-week-old WT and *Axin2^LacZ/LacZ^* mice by dividing the neogenic hair follicle count by the area of the wound containing neogenic hair follicles.

### Staining for AJ proteins and elastin in tissue sections of wounds

Healed wounds were harvested on PWD 14 from WT and *Axin2^LacZ/LacZ^* mice to generate formalin-fixed, paraffin-embedded blocks for sectioning. Antigen retrieval was performed on the deparaffinized tissue sections, which were then permeabilized using 0.1% Triton X-100.

To stain for AJ proteins, tissue sections were incubated overnight at 4°C using antibodies against E-cadherin [1:400, rabbit monoclonal antibody (mAb) no. 3195, Cell Signaling Technology] and β-catenin (1:100, Mouse mAb catalog no. 13-8400, Invitrogen). After incubation, the slides were washed three times in Dulbecco’s phosphate-buffered saline (DPBS) and then stained using secondary anti-mouse [1:500, Alexa Fluor 568 donkey anti-mouse immunoglobulin G (IgG), catalog no. A10037, Invitrogen] and anti-rabbit (1:500, Alexa Fluor 647 donkey anti-rabbit IgG, catalog no. A32795, Invitrogen) antibodies for 1 hour at room temperature. After two additional washes, glass coverslips were mounted onto each slide using a 4′,6-diamidino-2-phenylindole (DAPI)–containing mounting medium.

Confocal microscopy was carried out using the Leica Stellaris confocal microscope, and images were acquired as *Z*-stacks on LAS-X software. Images extracted from the *Z*-stack were analyzed to determine the fluorescence intensity of β-catenin expression on cell-cell junctions and to measure the width of cell-cell junctions expressing E-cadherin. For each condition, measurements from 10 to 20 cells were used to calculate the mean. The number of nuclei with positive β-catenin staining was counted manually.

To visualize the elastin fibers, Luna staining was carried out as previously described (*70*) at the Penn Skin Biology and Diseases Resource-based Center (SBDRC)’s Cutaneous Phenomics And Transcriptomics Core. After counterstaining using iron hematoxylin, glass coverslips were mounted onto each slide. The slides were scanned using the NanoZoomer 2.0-HT machine (Hamamatsu Photonics, Hechendorf, Germany) before image analysis.

### Live-cell imaging of the actin network in wound tissue

WT and *Axin2^LacZ/LacZ^* mice were wounded per the WIHN protocol. The healed wounds were harvested on PWD 14 and then washed in DPBS supplemented with penicillin, streptomycin, and amphotericin B (antibiotic-antimycotic).

The wounds were placed on 35-mm petri dishes with a glass bottom (no. 1.5 coverslip with a 20-mm glass diameter, catalog no. P35G-1.5-20-C, Mattek) and incubated overnight in cell culture media containing a fluorescent live-cell imaging probe for F-actin (100 nM SiR-actin, catalog no. CY-SC001, Cytoskeleton Inc., Denver, CO). The medium was supplemented with verapamil (10 μM), a broad-spectrum efflux pump inhibitor that improves probe retention, antibiotic-antimycotic, and 1:1000 dilution of NucSpot Live 488 (catalog no. 40081, Biotium, Fremont, CA)—a fluorescent nuclear dye suitable for live-cell imaging.

To image the epidermal portion of the healed wound, confocal microscopy was carried out using the Leica Stellaris confocal microscope (20× water immersion objective lens; numerical aperture, 0.75). Images were captured using Nyquist settings in photon counting mode as *Z*-stacks on LAS-X software, and image deconvolution was performed using Huygens Professional software.

To determine the fluorescence intensity of actin filaments, images extracted from the *Z*-stack were analyzed using ImageJ (blinded analysis using the Filename_Randomizer macro). From each wound, integrated density measurements of 50 by 50–pixel areas in the perinuclear cytoplasm were obtained from 25 to 52 cells in two to four *Z*-levels to calculate the reported mean values.

### qPCR analysis of wound tissue

On the day of scab detachment, mice were euthanized. Their re-epithelialized wounds were harvested and kept on ice. For qPCR detection of *Lef1*, lysates of these processed wound samples were prepared to extract their RNA (RNeasy Mini Kit, catalog no. 74004, Qiagen, Hilden, Germany) for conversion into cDNA using the High-Capacity cDNA Reverse Transcription Kit (catalog no. 4368814, Applied Biosystems, Waltham, MA).

The StepOnePlus Real-Time PCR System (Thermo Fisher Scientific) was used with primers (table S2) purchased from Integrated DNA Technologies (Coralville, IA). The difference in threshold cycle (Δ*C*_t_) for each target gene was calculated relative to that of *Gapdh* (housekeeping gene), and the fold change was calculated using the 2^−ΔΔ*C*t^ method.

### SHG

SHG images were obtained using unstained sections, cut from formalin-fixed, paraffin-embedded blocks. These blocks were generated using wounds harvested on scab detachment day from 3-week-old mice that underwent the WIHN protocol. Imaging was performed at the Penn Vet Imaging Core using a Leica TCS SP8 Multiphoton Confocal Microscope. The Coherent Chameleon Ultra II Ti:Sapphire laser (Coherent Inc., Santa Clara, CA) was tuned to 910 nm. The backward SHG signal was collected on a nondescanned hybrid detector configured to capture wavelengths at 455 nm (20× water immersion objective). From each image, three to four nonoverlapping areas of equal size (250 by 250 pixels) were extracted from the wound’s center, inner one-third, inner two-thirds, and periphery to undergo analysis by CT-FIRE, open-source software used to analyze collagen fiber properties (*71*). After extracting collagen fibers using CT-FIRE, the length, width, and collagen fiber density were calculated. For collagen orientation analysis, the dominant orientation and coherency of the collagen network were calculated using OrientationJ (*72*), an open-source plug-in for ImageJ (version 1.52a, National Institutes of Health).

### AFM measurements

AFM studies were conducted at the Scanning Local Probe Facilities using the Asylum MFP-3D (Asylum Research, Santa Barbara, CA) at the Singh Center for Nanotechnology at the University of Pennsylvania. Force measurements were obtained in contact mode within medium solution (calibration steps and sample measurements were performed in DPBS for tissue and tissue culture media for cells). Measurements were carried out at ambient temperature for tissue and 37°C for cells.

The probes (glass sphere probe or nanoneedle) were mounted on AFM cantilevers. For the spherical probe (Novascan Technologies, Boone, IA), the cantilever spring constant (*k*, ~0.03 N/m, calculated via the thermal tuning method) and the inverse of the optical lever sensitivity were calculated immediately before sample measurement for every experiment. For the nanoneedle (see the “Nanoneedle AFM” section), the spring constant was determined via the Asylum “Get Real” routine that combines Sader and thermal tuning methods in air. Optical sensitivity in situ was determined by thermal tuning in cell media, keeping *k* constant and solving for optical sensitivity.

To avoid the substrate effect (i.e., potential contribution to *E* from the underlying rigid substrate), all force measurements were obtained at low trigger (i.e., maximum) force [2 nN for both tissue and living cells, except for nanoneedle AFM studies that used a trigger force of 10 nN to ensure adequate membrane penetration (*33*)] to keep the indentation depth below 10 to 20% of sample thickness (*73*, *74*). Prior AFM indentation experiments of eukaryotic cells demonstrate that *E* measurements remain independent of indentation depth when deformation stays below 20% (*74*). This condition was met during our AFM measurements of tissue since the indentation depth remained below 90 nm (<1% of total tissue thickness) at a trigger force of 2 nN. For all cell measurements, the indentation depth was 0.1 to 2.3 (0.7 to 15.3% of keratinocyte height, 10 to 20 μm).

### Tissue preparation for AFM measurement of mouse tissue

Before harvest, the hair was clipped and depilated using Nair. Mouse skin was harvested down to the level of the subcutaneous fat after euthanasia. After subcutaneous fat removal using sharp forceps, the skin was laid flat on a transparent plastic plate and secured on all four corners using small magnets. Measurement sites were marked on the underside of the plastic plate using a black permanent marker and visualized during AFM using an inverted light microscope attached to Asylum MFP-3D. For each quadrant of the wound, AFM measurements were obtained at multiple points between the center and the wound periphery (see the schematic in fig. S2A). Surrounding normal skin measurements were taken >3 mm away from the wound edge. For each point, at least three consecutive force measurements were obtained. The reported *E* represents the mean value derived from technical replicate measurements.

### Wnt agonist treatment of keratinocytes and fibroblasts

Murine cells were collected from neonatal WT B6 pups (day 2) and processed to isolate epidermal keratinocytes and dermal fibroblasts, as previously described (*75*). At the Penn SBDRC’s Skin Translational Research Core, NHEK cells were isolated from neonatal foreskin samples collected from deidentified donors.

Keratinocytes were cultured in a 1:1 mixture of Keratinocyte-SFM containing l-glutamine (catalog no. 10724-011, Gibco) and Cascade Biologics Medium 154 (catalog no. M-154-500, Gibco). The medium was supplemented with human keratinocyte growth supplement (catalog no. S0015, Gibco), human recombinant epidermal growth factor (catalog no. 10450-013, Gibco), and bovine pituitary extract (catalog no. 13028-014, Gibco). Fibroblasts were cultured in Dulbecco’s modified Eagle’s medium with 10% fetal bovine serum, glucose (4.5 g/liter), l-glutamine, and sodium pyruvate (catalog no. 50-003-PC, Corning, Corning, NY). The cell culture medium was supplemented with penicillin, streptomycin, and amphotericin B (antibiotic-antimycotic, catalog no. 15240096, Gibco).

Cells were plated onto a commercially available 35-mm petri dish (made-to-order CytoSoft Rigidity Plates from Advanced Biomatrix, Carlsbad, CA)—containing a uniform, thin layer of silicone (0.5 mm) of known rigidity (8 or 32 kPa)—or a standard dish composed of tissue culture plastic. Before plating, dishes were pretreated for 30 min with type I bovine collagen solution (0.1 mg/ml; Purecol Bovine Collagen, 3 mg/ml, catalog no. 5005, Advanced Biomatrix) to facilitate the seeding process. A sufficient volume was used to cover the entire dish. After overnight incubation, the cells were treated for 24 to 48 hours as follows: CHIR99021 (catalog no. 4423, Tocris Bioscience, Bristol, UK) administered at 1.5, 3, or 6 μM; LiCl (catalog no. L9650, Millipore Sigma, Burlington, MA) administered at 10 mM; and recombinant human DKK4 (catalog no. 1269DK010, R&D Systems, Minneapolis, MN) administered at 0.5 to 1 μg/ml.

### AFM of live cells using a spherical probe

Cell-containing regions of the dish were identified using an inverted light microscope attached to Asylum MFP-3D, and consecutive force measurements were obtained at 37°C to construct a force map (6 by 6 grid, 25 μm in each dimension). The AFM tip was directly visualized in real time using an inverted microscope to allow precise selection of measurement sites. For each experimental condition, at least five force maps were obtained and analyzed to calculate *E*.

### Nanoneedle AFM

#### Microfabrication of nanoneedles

Dual-beam plasma focused ion beam milling and scanning electron microscopy were performed with a Xe plasma Tescan S8252X on MikroMasch CSC12 350-μm by 35-μm silicon probes (shape, pyramid; MikroMasch, Tallinn, Estonia) with Cr-Au coating (nominal *k* = 0.05 N/m). Initial trenching was done with a 30-kV, 1-nA beam, intermediate shaping at 30 kV and 100 pA, and final polishing at 30 kV and 10 pA. Scanning electron microscopy images of the probe were obtained before, during, and after the milling (time-lapse video of the milling process shown in movie S1). For the nanoneedle used to obtain intracellular measurements, the base width was 250 nm at a 1.4-μm height (away from tip) and 408 nm at a 3-μm height.

#### Nanoneedle AFM

Nanoneedle AFM was performed using NHEK cells grown in a monolayer after treatment with 6 μM CHIR for 48 hours. Cells were cultured on 35-mm petri dishes with a glass bottom (no. 1.5 coverslip with a 20-mm glass diameter, catalog no. P35G-1.5-20-C, Mattek, Ashland, MA) to directly visualize subcellular compartments (nuclei, cytoplasm, and actin-rich cell-cell junctions) in real time. To guide site selection, bright-field imaging was coupled with fluorescence microscopy using live-cell stains against nuclei (1:1000 dilution of NucSpot Live 488) and F-actin (100 nM SiR-actin). Before nanoneedle AFM, the cells were incubated overnight in media containing these probes and 10 μM verapamil. Each measurement site was first selected using bright-field microscopy and then confirmed using fluorescence microscopy.

Intracellular AFM measurements were obtained at 37°C using a nanoneedle probe. A trigger force of 10 nN with a tip velocity between 8 and 10 μm/s was used to achieve membrane penetration (*33*). The width of the nanoneedle did not exceed 400 nm for cell penetration depths (<2.3 μm) seen during nanoneedle AFM. As described in previously validated methods (*32*–*34*), successful cell membrane penetration was visually confirmed on the force-distance plot through the identification of a local force peak—characterized by a steep increase around 0.5 to 1 μm of indentation during the approach phase, followed by an incremental decline (fig. S6). For measurement sites overlying a nucleus, a second peak between the first peak and the transition point was used to signify nuclear membrane penetration. Liu *et al*. previously established that these force peaks were reliable indicators of successful membrane penetration using reconstructed *Z*-stack confocal images simultaneously obtained during AFM (*33*). To exclude measurements taken from cells with incomplete cell/nuclear membrane penetration, only force-distance curves with an appropriate number of clearly defined peaks were analyzed.

### Time-resolved AFM

NHEK cells were grown in monolayer 35-mm petri dishes (substrate *E* = 32 kPa) and then treated with 6 μM CHIR for 24 hours. Cells were treated with 1 mM EDTA (UltraPure 0.5 M EDTA, pH 8.0, catalog no. 15575020, Gibco) in calcium-free media (KGM 2 without Ca++ BulletKit Medium-2, catalog no. CC-3108, Lonza) for 30 min. After two washes with DPBS with no calcium or magnesium, calcium-containing medium was reintroduced. For the treatment group, CHIR was reintroduced with a final concentration of 6 μM. Serial force maps were obtained immediately after treatment over nonoverlapping regions of the dish, separated by at least 2 to 3 mm from one another, for 30 min in 3-min intervals.

### Young’s modulus calculation from AFM data

For all AFM studies, *E* was calculated from the force-distance curves using AtomicJ, an open-source analysis software (*76*). Calculation parameters for appropriate contact models based on tip geometry (cone for nanoneedle AFM of live cells and sphere for all other AFM studies) are as follows.

#### Sphere

To account for adhesion (i.e., van der Waals forces) between the sample and the probe, the Derjaguin-Muller-Toporov model of elastic contact with Maugis’ correction (*77*) was used

$$P=\frac{4E\sqrt{R}}{3\left(1-\nu^{2}\right)}\;\delta^{3/2}-2\pi \gamma R$$

*P* is the load, *E* is Young’s modulus, *R* is the probe’s apex tip radius (2.5 μm), ν is Poisson’s ratio (0.5), γ is the work of adhesion, and δ is the indentation depth.

The contact model was fitted to the force-indentation data between the contact point, where the probe contacts the sample and causes an elevation in force, and the transition point, where the trigger force was met and causes the cantilever to retract.

#### Cone

$$P=\frac{2E\;\text{tan}\left[\theta \right]}{\pi \left(1-\nu^{2}\right)}\;\delta^{2}$$

*P* is the load, *E* is Young’s modulus (kPa, 1 kPa = 1000 N/m^2^), δ is the indentation depth, ν is Poisson’s ratio, and θ is the half angle of the probe. Poisson’s ratio of 0.5 was used (*78*). The half angle of the nanoneedle probe (θ = 3.9°) was measured via ImageJ using scanning electronic microscopy images taken before AFM measurements.

### PZHR area calculation

The area of PZHR (i.e., wound area with optimal tissue rigidity for WIHN, *E* between 5 and 15 kPa) was calculated as follows. For each experimental group, AFM-derived *E* values for each measurement site (center, inner one-third, inner two-thirds, and periphery) on PWD 14 were fitted to a polynomial regression model (second order) using the least-squares method. Measurement sites were converted to numerical values as a proportion of the length between the wound center and the periphery as follows: center (0), inner one-third (0.33), inner two-thirds (0.67), and periphery (*1*). Using the resulting regression equations, *X*-intercept values for 15 kPa (*X*_15kPa_) were calculated (see fig. S2, A and B). Since *E* exceeded 5 kPa on all measurement sites, the PZHR area was calculated as *A* = (*X*_15kPa_)^2^. Data analysis, including goodness-of-fit calculations (*R*^2^), was performed using GraphPad Prism.

### Capillary electrophoresis immunoassay (Simple Western) and qPCR analysis of NHEK cells

CHIR-treated NHEK keratinocytes were grown in a monolayer on 35-mm tissue culture plastic petri dishes and treated with CHIR (3 and 6 μM for qPCR and 6 μM for Simple Western) for 48 hours. After treatment, cells were harvested and lysed for further processing.

RNA was extracted using the RNeasy Mini Kit and converted to cDNA using the High-Capacity cDNA Reverse Transcription Kit to analyze mechanotransduction and canonical Wnt signaling pathways (primers listed in table S2). Δ*C*_t_ for each target gene was calculated relative to that of *GAPDH* (housekeeping gene), and the fold change was calculated using the 2^−ΔΔ*C*t^ method.

For Simple Western analysis of β-catenin in cytoplasmic and membrane fractions, subcellular fractionation was performed using a commercially available kit (Subcellular Protein Fractionation Kit for Cultured Cells, catalog no. 78840, Thermo Fisher Scientific).

The Simple Western assay was performed per the manufacturer’s instructions using Jess (Bio-Techne, Minneapolis, MN) with the following consumable reagents: 12- to 230-kDa Separation Module (catalog no. SM-W001, Bio-Techne) and anti-Rabbit Detection Module (catalog no. DM-001, Bio-Techne). Antibodies used are listed as follows: E-cadherin (Rabbit mAb no. 3195, Cell Signaling Technology), β-catenin (Rabbit mAb no. 9582, Cell Signaling Technology), GAPDH (glyceraldehyde-3-phosphate dehydrogenase; Rabbit polyclonal NB100-56875, Novus Biologicals), and Na,K-ATPase α1 (Rabbit mAb no. 23565, Cell Signaling Technology). ProteinSimple Compass software (Bio-Techne) was used for analysis. Unprocessed blots are provided (fig. S11).

### Staining for cell junction proteins and actin in NHEK cells

NHEK cells grown on glass coverslips were fixed in 4% paraformaldehyde (Affymetrix, Santa Clara, CA) for 15 minutes and washed twice using DPBS.

#### Phalloidin staining

Cells were permeabilized with 0.1% Triton X-100 for 15 min, washed twice with DPBS, stained using Alexa Fluor 488 Phalloidin (catalog no. A12379, Invitrogen) for 30 min, and mounted on a glass slide using a DAPI-containing mounting medium.

#### Staining for E-cadherin, β-catenin, and desmogleins 1/3

The fixed cells were incubated overnight in 4°C using antibodies against E-cadherin (1:400, Rabbit mAb no. 3195, Cell Signaling Technology), β-catenin (1:500, Mouse mAb catalog no. 13-8400, Invitrogen), or desmogleins 1/3 (Px44, a gift from A. Payne and X. Mao). Px44, monoclonal anti-desmoglein single-chain variable fragment antibodies, were previously generated by isolating human anti-desmoglein mAbs from a donor patient with mucocutaneous pemphigus vulgaris (*79*).

After incubation, the fixed cells were washed three times in DPBS and then stained using secondary anti-human (1:500, Alexa Fluor 594 goat anti-Human IgG, catalog no. 109-585-003, Jackson ImmunoResearch), anti-mouse (1:500, Alexa Fluor 488 donkey anti-mouse IgG, catalog no. A-21202, Invitrogen), or anti-rabbit (1:500, Texas Red goat anti-Rabbit IgG, catalog no. T-2767, Thermo Fisher Scientific) antibodies for 1 hour at room temperature. Glass coverslips were mounted onto each slide using a DAPI-containing mounting medium.

Confocal microscopy was performed using the Leica TCS SP8 WLL scanning laser confocal microscope, equipped with HyD detectors in sequence, on Leica LAS-X software (Leica Microsystems, Buffalo Grove, IL). Images were acquired as *Z*-stacks using a 63×/1.40 oil objective, with 2× zoom and a pinhole of 1 Airy unit, with an average of 35 *z*-steps through 10.5 μm, to yield a voxel size of 90 by 90 by 299 nm. Postprocessing was performed using LAS-X to generate 3D images for actin staining and 2D images (maximum intensity projection) for actin, E-cadherin, desmogleins, and β-catenin staining.

### Staining for adhesion zipper proteins in NHEK cells

NHEK cells were plated at ~30% confluency on 35-mm petri dishes with a glass bottom and treated with 6 μM CHIR for 1, 3, 6, or 24 hours before harvesting. Harvested cells were permeabilized with 0.1% Triton X-100 for 15 min, washed twice with DPBS, and incubated overnight at 4°C with antibodies against E-cadherin (1:400, Rabbit mAb no. 3195, Cell Signaling Technology), vinculin (1:500, Rat mAb no. 938402, BioLegend, San Diego, CA), and VASP (1:50, Mouse mAb no. 610448, BD Transduction Laboratories).

Confocal imaging was carried out using the Leica Stellaris confocal microscope (40× water immersion objective lens; numerical aperture, 1.1). Images were acquired as *Z*-stacks on LAS-X software, and postprocessing was performed on ImageJ to generate maximum-intensity-projection images for analysis.

### Staining for phospho-MLC2 and actin in NHEK cells

NHEK cells were plated on 35-mm petri dishes with a glass bottom and treated with 6 μM CHIR for 48 hours before harvesting. Cells were permeabilized with 0.1% Triton X-100 for 15 min, washed twice with DPBS, stained using Alexa Fluor 488 Phalloidin for 30 min at room temperature, and then incubated overnight in 4°C using antibodies against phospho-MLC2 at serine-19 (1:50, Rabbit mAb no. 3671, Cell Signaling Technology). Subsequently, the cells were washed three times in DPBS and stained using secondary anti-rabbit (1:500, Alexa Fluor 594 goat anti-rabbit IgG, catalog no. A-11012, Invitrogen) antibodies for 1 hour at room temperature before mounting using a DAPI-containing mounting medium.

Confocal imaging was carried out using the Leica Stellaris confocal microscope (40× water immersion objective lens, 2× zoom). Images were acquired as *Z*-stacks on LAS-X software, and postprocessing was performed to generate maximum-intensity-projection images for analysis.

### Analysis of confocal microscopy images

Using 3D images of actin, CT-FIRE was used to measure the length and width of extracted individual actin fibers. The coherency of junctional actin filaments was measured using the “Measure” feature on OrientationJ, which was also used to create images in HSB mode. The mean integrated density of fluorescence was measured for both 2D and 3D images of actin using ImageJ.

Nuclear translocation of β-catenin was measured using JACoP (*80*), an ImageJ plug-in. Manders’ coefficient (M1) was calculated using a preset threshold to quantify the degree of colocalization between DAPI and β-catenin (0, no colocalization; 1, perfect colocalization).

Membrane E-cadherin expression was quantified as follows. On ImageJ, the maximum-intensity-projection images were converted to a grayscale 8-bit format. For each cell, the fluorescence intensity profile of a line drawn between the cytoplasm and the cell membrane was analyzed (see Fig. 4G). For each condition, three separate fields were analyzed. Corrected total cell fluorescence measurements [integrated density − (area × mean background fluorescence)] for E-cadherin were also calculated using ImageJ.

To determine the fluorescence intensity of phospho-MLC2, maximum-intensity-projection images were analyzed using ImageJ. From each condition, integrated density measurements of 10 by 10–pixel areas from the cytoplasm and cell-cell junctions were obtained from 100 to 110 cells (three separate dishes, at least three fields per dish) to calculate the reported values.

### FLIM-FRET analysis

NHEK cells were cultured on 35-mm petri dishes with a glass bottom and treated with 6 μM CHIR. After 48 hours of treatment, cells were harvested, permeabilized with 0.1% Triton X-100 for 15 min, washed twice with DPBS, and incubated overnight at 4°C with antibodies against the cytoplasmic domain of E-cadherin (1:400, Rabbit mAb no. 3195, Cell Signaling Technology) and the 100 C-terminal residues of β-catenin (1:500, Mouse mAb catalog no. 13-8400, Invitrogen). Notably, the interaction surface between E-cadherin and β-catenin is extensive and involves the 100 C-terminal residues of β-catenin (*50*).

After overnight incubation, cells were washed twice with DPBS and incubated for 1 hour at room temperature with fluorescent dye–conjugated secondary antibodies to fluorescently label β-catenin (FRET donor, Alexa Fluor 488) and E-cadherin (FRET acceptor, Alexa Fluor 555). The following antibodies were used: donkey anti-mouse IgG conjugated with Alexa Fluor 488 (1:500, catalog no. A-21202, Invitrogen) and donkey anti-rabbit IgG conjugated with Alexa Fluor 555 (1:500, catalog no. A31572, Invitrogen). A schematic of FLIM-FRET studies is shown in fig. S9 (A and B).

FLIM-FRET acquisition and analysis were performed using a Leica Stellaris 8 FALCON confocal/FLIM microscope equipped with a 63× water immersion objective lens (numerical aperture, 1.2) at confluent portions of the dish. The FLIM data were acquired on LAS-X software (version 4.6.1.27508) with the excitation wavelength set to 488 nm and emission collected in the range of 493 to 523 nm. The optical zoom was set to 1.5× with 12 line and 10 frame repetitions at a format of 512 by 512 and a scan speed of 200 Hz. Appropriate control dishes labeled with the donor fluorophore only (i.e., cells stained with fluorescent antibodies against β-catenin but not E-cadherin) were included.

To measure the FRET efficiency, the average donor lifetime in the absence of an acceptor was first obtained using measurements taken from control dishes labeled with the donor fluorophore only. These measurements showed comparable values from CHIR-treated and untreated groups (~2.97 ns). Using the fluorescence lifetime images of cells labeled with both donor and acceptor fluorophores, regions of interest were selected from membrane regions (i.e., cell-cell interfaces), and the respective decay curves were fitted using a dual exponential fit on LAS-X software to calculate the FRET efficiency.

### TFM

Commercially available polyacrylamide-based hydrogels bound to six-well glass-bottom plates were purchased (Softwell 6 Glass, catalog no. SW6G, Matrigen, Irvine, CA). Each plate contained hydrogels with known substrate rigidities (12, 25, and 50 kPa). Fluorescent microspheres (excitation/emission, 580/605 nm) were bound to the hydrogel surface, which was coated with rat tail collagen I. Two sets of plates were prepared for each substrate rigidity. Plates were seeded with NHEK cells at 60,000 cells per well (5% confluence for single-cell TFM) or 360,000 cells per well (30% confluence for multicellular TFM) and then treated with LiCl (10 mM) for 48 hours before TFM. Before and after trypsinization (0.25% trypsin with EDTA), images were taken on Nikon NIS-Elements using the Nikon Eclipse Ti inverted microscope equipped with an enclosed chamber (5% CO_2_ at 37°C) for live-cell imaging. Imaging was performed at the Wistar Institute’s Imaging Facility.

Live TFM analysis was performed by comparing the topmost bead planes underlying the cells at the initial time point to the later time points. Images were aligned for drift correction using the normalized cross-correlation algorithm (Fiji plug-in: Align Slices in Stack). Drift-corrected images were used to calculate the bead displacement field $\left[\underset{u}{\to }\left(\underset{r}{\to }\right)\right]$ using a custom particle image velocimetry software (*81*). Parameters were three interrogation windows of 64, 32, and 16 pixels. Traction forces $\left[\underset{T}{\to }\left(\underset{r}{\to }\right)\right]$ for single cells were calculated from the displacement fields using regularized Fourier transform traction cytometry with Poisson’s ratio of 0.5 and a regularization factor of $10^{-7}$, as previously described (*82*).

For cell colonies, the traction field was calculated by solving the inverse problem using the nonregularized Fourier transform algorithm previously used by Trepat *et al.* (*83*). On the basis of traction forces, we calculated strain energy *U* as described by Butler *et al.* (*84*) per the equation below. All methods were implemented using custom MATLAB scripts

$$U=\left(\frac{1}{2}\right)\int \underset{T}{\to }\left(\underset{r}{\to }\right).\underset{u}{\to }\left(\underset{r}{\to }\right)\text{dxdy}$$

### STORM

Commercially available polyacrylamide-based hydrogels bound to 24-well polystyrene plates were purchased (Softslip 24, catalog no. SS24, Matrigen). Each plate contained hydrogels with known substrate *E* (8 and 25 kPa) that were surface coated with rat tail collagen I. Plates were seeded with NHEK cells at 60,000 cells per well and then treated with CHIR (3 or 6 μM) or LiCl (10 mM) for 48 hours. Cells were fixed using 4% paraformaldehyde, permeabilized with 0.5% (v/v) Triton X-100 (Sigma-Aldrich) for 15 min, and blocked with 3% (w/v) BSA (Sigma-Aldrich) for 1 hour. Samples were then stained overnight at 4°C with H2B primary antibody (ProteinTech no. 15857-1-AP, 1:50) and secondary antibodies custom labeled with activator-reporter dye pairs [Alexa Fluor 405 (A30000)-Alexa Fluor 647 (A20006), Invitrogen] for STORM imaging, as previously described (*85*). All images were taken using a commercial STORM microscope system from ONI (Nanoimager S) fitted with a ×100, 1.4–numerical aperture oil-immersion objective and a sCMOS Hamamatsu Ocra Flash camera. A previously described imaging buffer was used to promote proper photoswitching of Alexa Fluor 647 [10 mM cysteamine MEA (Sigma-Aldrich, 30070-50G) in Glox Solution: 1-glucose oxidase (0.5 mg/ml), 1-catalase (40 mg/ml) (both Sigma-Aldrich), and 10% glucose in PBS] (*86*, *87*). The 640-nm laser (~1000-mW peak power) was used at a setting of 40% to excite the reporter dye (Alexa Fluor 647, Invitrogen) and switch it to the dark state for 10k frames, and the 405-nm laser power was then gradually increased to reactivate Alexa Fluor 647 in an activator dye (Alexa Fluor 405)–facilitated manner to maintain a constant density of active fluorophores. A total of 30,000 frames were recorded per image at 15-ms exposure per frame. STORM image localizations were compiled using Nanoimager software (ONI).

For quantitative analysis, nuclei were manually cropped from the images and custom-written MATLAB codes were used to perform Voronoi tessellation of the fluorophore localizations (*88*, *89*). Voronoi tessellation assigns a Voronoi polygon to each localization such that the polygon area is inversely proportional to the local localization density (*90*). To quantify the relative level of chromatin compaction in nuclei under different treatments, a Voronoi polygon area threshold was applied to the set of localizations from each nucleus to distinguish denser heterochromatic portions of the chromatin from less dense euchromatic regions corresponding to regions of denser and sparser spatial distributions of localizations, respectively. Localizations with Voronoi polygons with areas smaller than the threshold (corresponding to regions of high localization density) were classified as heterochromatin. The threshold was chosen such that, on average, ~50% of the localizations in the 8-kPa control nuclei were classified as belonging to the heterochromatic fraction. This same threshold was then applied to all treatment conditions to allow for relative comparisons.

Clustering using MATLAB’s DBSCAN algorithm (*91*) was performed on the localizations within the heterochromatic fraction to identify individual subdomains, which include all neighboring and connected Voronoi polygons below the area threshold. The approximate radii of each of these subdomains were measured by using the outer extent of the included localizations to define a polygon, finding the area of this polygon, and then calculating *r* ≈ √Domain area/π, assuming a roughly circular shape. Chromatin clusters obtained after the DBSCAN subdomain classification were categorized as peripheral and nonperipheral chromatin depending on proximity to the boundary of the nucleus image. The nucleus shape was detected using the boundary-detecting algorithm, and a characteristic radius of the nucleus (*R*) was then calculated. The minimal distance between each heterochromatin subdomain and nucleus boundary was calculated, such that any subdomain having a distance smaller than 0.15*R* was classified as part of the peripheral domain (*92*). The local peripheral chromatin thickness was measured by sampling the boundary along the nucleus periphery.

### Collective durotaxis assessment

Two-layered polyacrylamide hydrogels (acrylamide monomer concentrations of 20 and 4%) with a linear stiffness gradient (8.2 kPa/mm; stiffness range, 0 to 164 kPa) were generated on 20-mm glass coverslips using the double polymerization method described previously (*93*). Gels were purchased from Matrigen as a custom order. The presence of a linear stiffness gradient was confirmed using AFM. For live-cell imaging, hydrogels were glued to 35-mm petri dishes.

Small monolayers of NHEK cells were created at local regions of the gel with a substrate rigidity of ~30 kPa (3.6 mm from the soft end of the gel) by dispensing 8-μl drops of highly concentrated solution of cells (~5.9 × 10^6^ to 6.7 × 10^6^ cells/ml). After incubation for 1 hour at 37°C, unattached cells were aspirated, and the gel surface was washed twice using DPBS. The medium was added to cover the entire gel surface. After 24 hours, cells were incubated with CHIR (6 μM) overnight.

Nuclei were visualized using Hoechst 33342 (NucBlue Live ReadyProbes Reagent, catalog no. R37605, Invitrogen), and live-cell imaging was performed at the Wistar Institute’s Imaging Facility. Serial 5× images were obtained over 12 hours at 30-min intervals on Nikon NIS-Elements using the Nikon Eclipse Ti inverted microscope equipped with an enclosed chamber (5% CO_2_ at 37°C).

Image stacks were processed using Fiji, and fluorescent nuclei were tracked using TrackMate—a free ImageJ plug-in for single-particle tracking (StarDist used for nuclear detection) (*94*). Mean displacement and mean velocity for all cells in a field were analyzed using the “track mean speed” and “total distance traveled” parameters from TrackMate.

### Statistics and reproducibility

Data analysis and graph generation were performed using GraphPad Prism 10 (GraphPad Software, San Diego, US). Unless stated otherwise, data are presented as means ± SEM. For box-and-whisker plots, the central line represents the median, the box represents the interquartile range [25th (Q1) to 75th (Q3) percentile], and whiskers represent 10th and 90th percentiles.

Statistical significance was analyzed as follows. When two conditions were present, the significance for pairwise comparisons was assessed using a two-tailed unpaired *t* test [Figs. 1, F and G; 2, B (right), C, D, F, and H; 3, D and E; 4C; and 6, D to F; and figs. S1, A and B; S2C; S4, A, B, F, H, and J; S6, C and D; and S9D]. When three or more conditions were present with one independent variable, one-way analysis of variance (ANOVA) was used with Tukey’s multiple comparisons test [Figs. 1, C to E; 2, A and B (left); 3, J and K; and 4E and figs. S1, C and D, S2F, and S4C). When three or more conditions were present with two independent variables, two-way ANOVA was conducted with Tukey’s multiple comparisons test (Fig. 5, B and D; and figs. S4D, S5B, S7C, and S10, B to D). For experiments with repeated measurements, two-way repeated-measures ANOVA was used (Fig. 2J). Multiple unpaired *t* tests with the two-stage step-up method of Benjamini, Krieger, and Yekutieli (desired *Q* = 1.00%) were carried out for Fig. 3 (F and G).

GraphPad Prism was also used to perform the regression analysis (Figs. 1, D and E, and 4D) and to calculate skewness (Fig. 6C). The sample size reported in mouse studies refers to biological replicates. The sample size reported in imaging studies and in vitro studies (i.e., fields, force maps etc.) refers to technical replicates, unless otherwise specified. All experiments (Figs. 1 to 7) were repeated at least once.

Statistically significant differences were considered when *P* < 0.05 and were denoted as follows: *P < 0.05, **P < 0.01, ***P < 0.001, and ****P < 0.0001.
