# Supplementary material for: Wnt signaling modulates mechanotransduction in the epidermis to drive hair follicle regeneration
Source: Sci Adv. 2025 Feb 19;11(8):eadq0638. doi: 10.1126/sciadv.adq0638 (PMC11838001; doi:10.1126/sciadv.adq0638)
Supplement: Supplementary file 1 — Figs. S1 to S11 Tables S1 and S2 Legend for movie S1 [file sciadv.adq0638_sm.pdf]

Science Advances
AAAS

Supplementary Materials for

Wnt signaling modulates mechanotransduction in the epidermis to drive hair follicle regeneration

Allen S. W. Oak *et al.*

Corresponding author: George Cotsarelis, cotsarel@pennmedicine.upenn.edu

Sci. Adv. **11**, eadq0638 (2025)
DOI: 10.1126/sciadv.adq0638

The PDF file includes:

Figs. S1 to S11
Tables S1 and S2
Legend for movie S1

Other Supplementary Material for this manuscript includes the following:

Movie S1

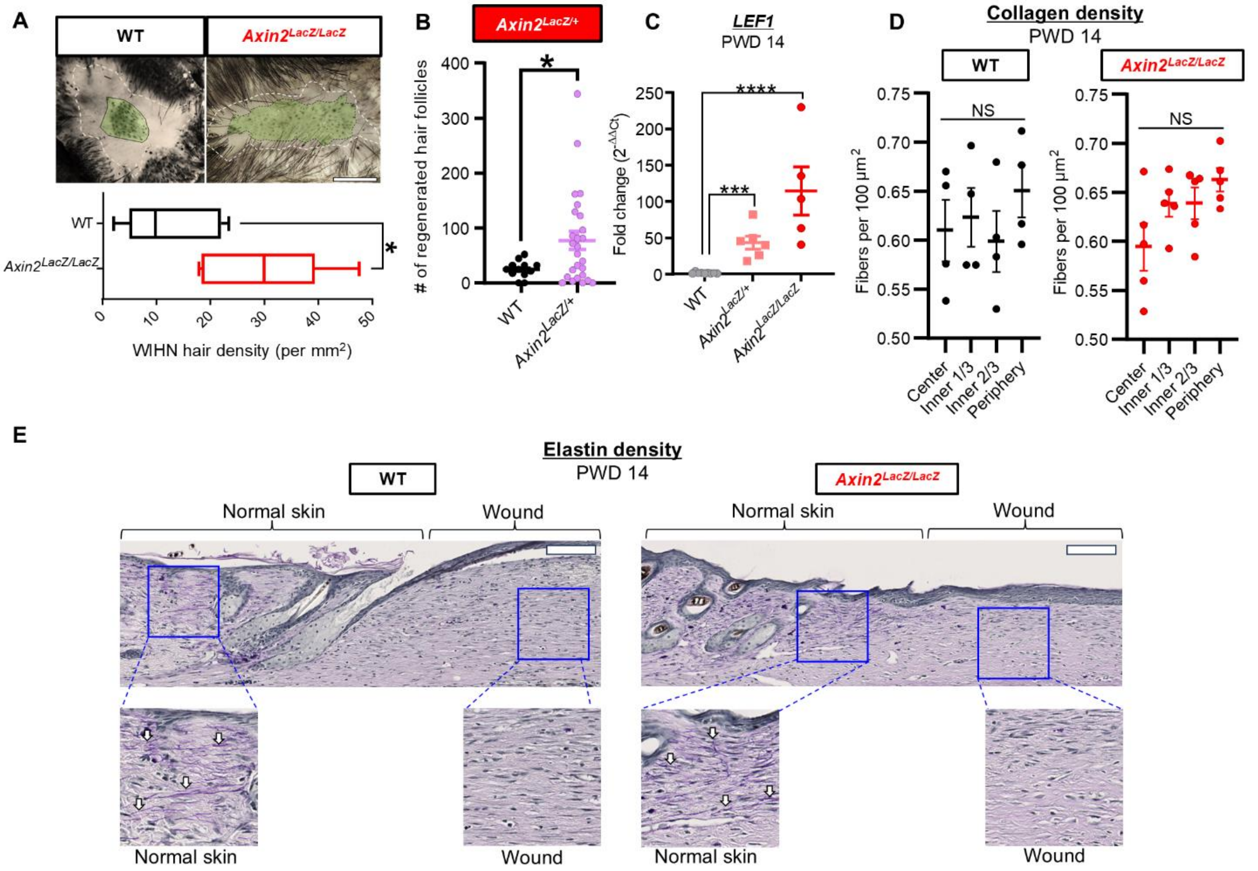

Fig. S1. Transgenic mice with amplified canonical Wnt signaling demonstrate increased WIHN. (**A**) Density of regenerated hair follicles is elevated in *Axin2*$^{LacZ/LacZ}$ mice compared to that of WT control group. *n*=5-6 mice per group. Representative images of AP staining on top. Scale, 2 mm. **(B and C)** Amplified Wnt signaling in *Axin2*$^{LacZ/+}$ heterozygote mice. (**B**) Elevated number of regenerated hairs seen in *Axin2*$^{LacZ/+}$ compared to WT mice (*n*=12-26 mice per group). AP staining performed on PWD 30. (**C**) qPCR for *LEF1* in wounds harvested during scab detachment. *LEF1* expression in *Axin2*$^{LacZ/+}$ mice was between those of WT and *Axin2*$^{LacZ/LacZ}$ mice (*n*=5-9 mice group). (**D**) SHG microscopy of wounds revealed similar collagen density throughout the wound in WT and *Axin2*$^{LacZ/LacZ}$ mice on the day of scab detachment (*n*=4-5 mice per group). (**E**) Representative images of Luna staining in wounds harvested on PWD 14. Sections were counterstained using iron hematoxylin. In normal skin adjacent to the wound edge (insets on left side), Luna staining revealed dermal elastic fibers highlighted in purple (white arrows). However, the healed wounds (insets on right side) showed little to no elastin fibers in both WT and *Axin2*$^{LacZ/LacZ}$ mice. Scale, 100 µm. Data represented as mean $\pm$ s.e.m. Box and whisker plot*:* central line, median; box, IQR (25th (Q1)–75th (Q3) percentile); and whiskers, 10th and 90th percentile. *$P < 0.05$; ***$P < 0.001$; ****$P < 0.0001$.

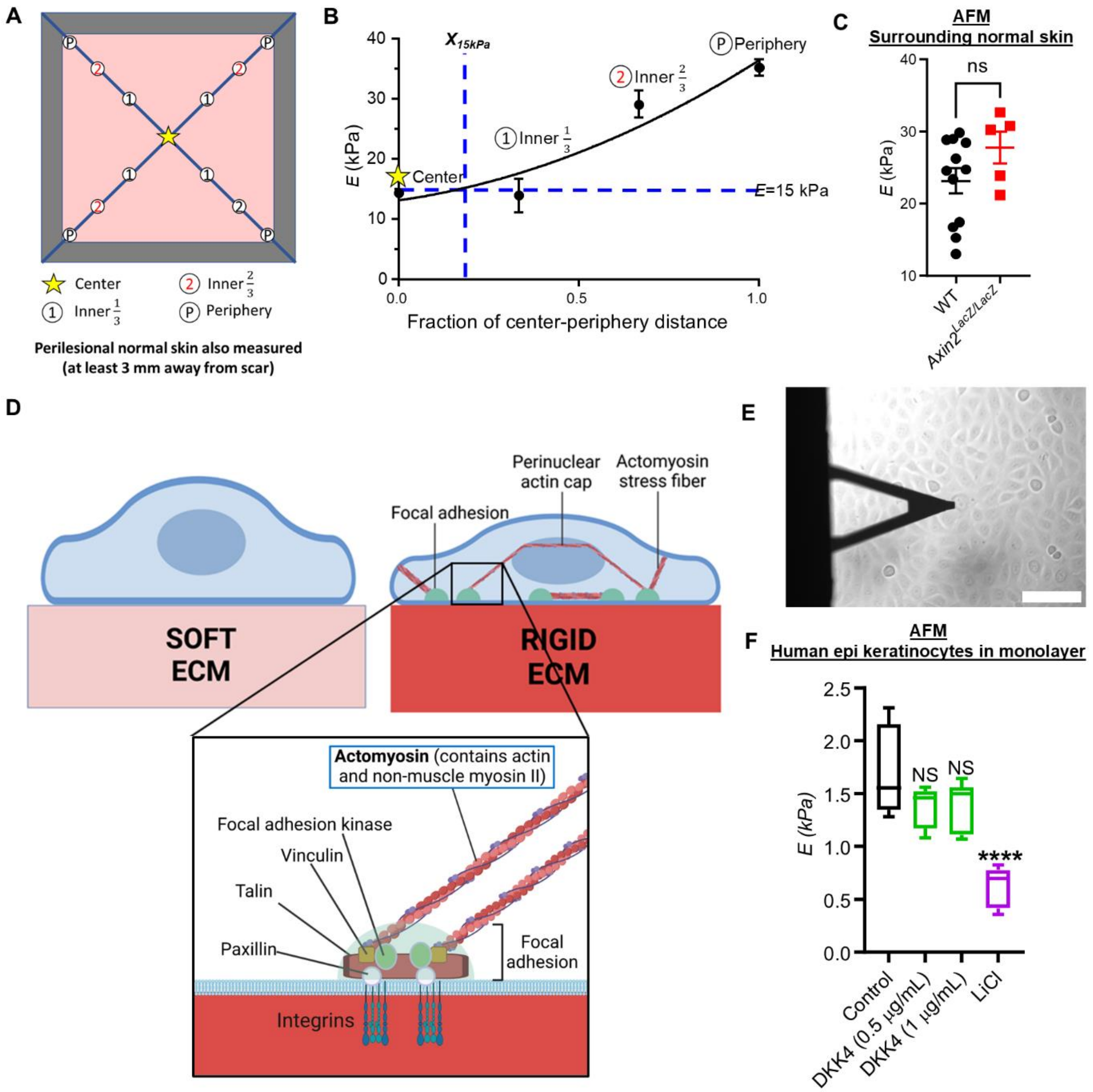

Fig. S2. Assessment of mechanotransduction using AFM. (**A**) AFM measurement sites on day of scab detachment. (**B**) Calculation of PZHR area. AFM measurement sites were converted to numerical values as fractions of the center-periphery distance (*i.e.*, center=0, inner 1/3=0.33, inner 2/3=0.67 and periphery=1) and plotted on the x-axis. On the y-axis, AFM-derived E values (represented as mean $\pm$ s.e.m.) were plotted for each group. Quadratic regression analysis was then performed to calculate the regression equation. Using the regression equation, x-intercept value for 15 kPa ($X_{15\text{kPa}}$,) was calculated. Since mean E exceeded 5 kPa on all measurement sites, PZHR area was calculated as $A = (X_{15\text{kPa}})^2$. (**C**) E of normal skin surrounding the wound. AFM measurements from the unwounded skin (> 3 mm away from wound edge on PWD 14) revealed no difference between WT (n=12 mice) and *Axin2*$^{LacZ/LacZ}$ (n=5 mice) mice. (**D**) Mechanotransduction-driven substrate rigidity response. Elevated ECM rigidity induces integrin clustering, which triggers a rapid self-assembly of mechanosensitive proteins to form the focal adhesion complex on cell membrane. The focal adhesion complex, containing talin and other

mechanosensitive proteins, recruits actomyosin bundles that contract to increase cell stiffness. **(E)** Representative field view during AFM measurement of human keratinocytes grown in monolayer. Regions with no visible gaps between cells were identified and force maps were obtained. Scale, 50 μm. **(F)** Suppression of Wnt signaling in human keratinocytes does not lead to cell stiffening. AFM was used to assess the substrate rigidity response in human keratinocytes grown in monolayer (substrate E = 32 kPa) and treated with Wnt agonist LiCl (10 mM) or Wnt antagonist DKK4 (0.5 or 1 μg/mL) for 24 hours. Cells treated with LiCl demonstrated lower cell rigidity values, but cells treated with DKK4 exhibited no change in their rigidity response. n=5-7 force maps per condition. Data represented as mean ± s.e.m. Box and whisker plots: central line, median; box, IQR (25th (Q1)–75th (Q3) percentile); and whiskers, 10th and 90th percentile. ****$P < 0.0001$.

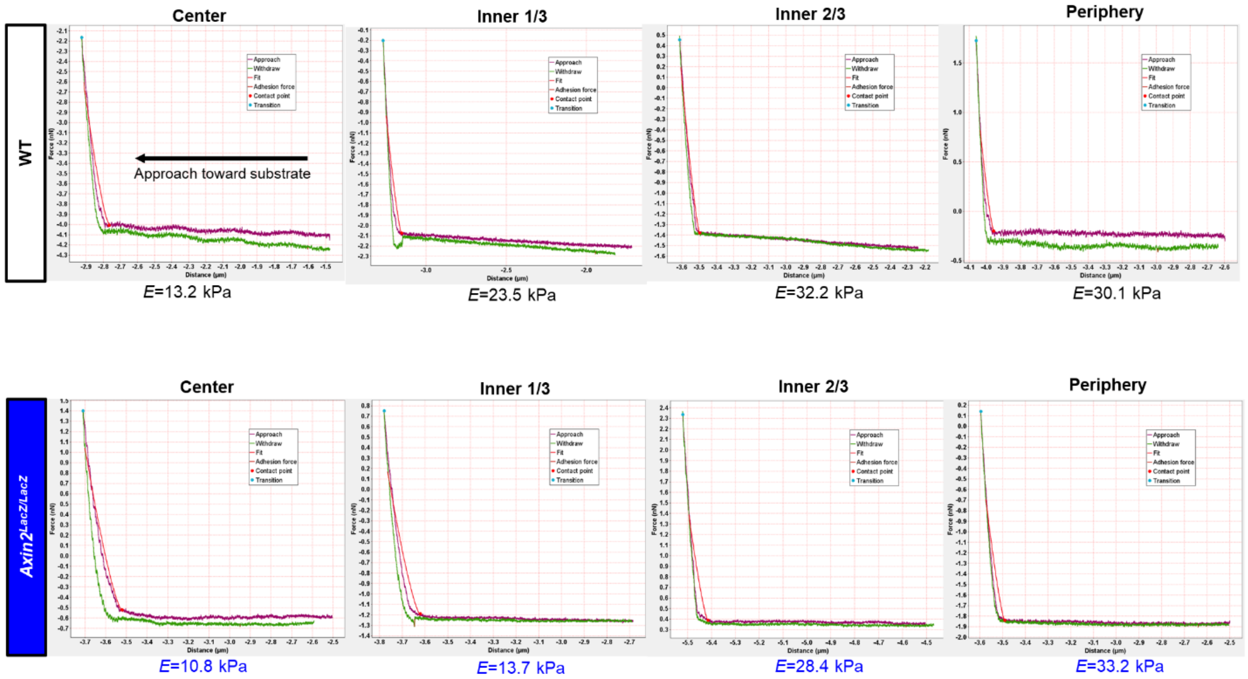

Fig. S3. Representative force-distance curves from AFM measurements of wounds on day of scab detachment. AFM measurements were obtained from the center, inner 1/3, inner 2/3 and periphery of wound in WT and *Axin2*$^{LacZ/LacZ}$ mice. Listed *E* values were calculated using the Derjaguin-Muller-Toporov (DMT) model of elastic contact.

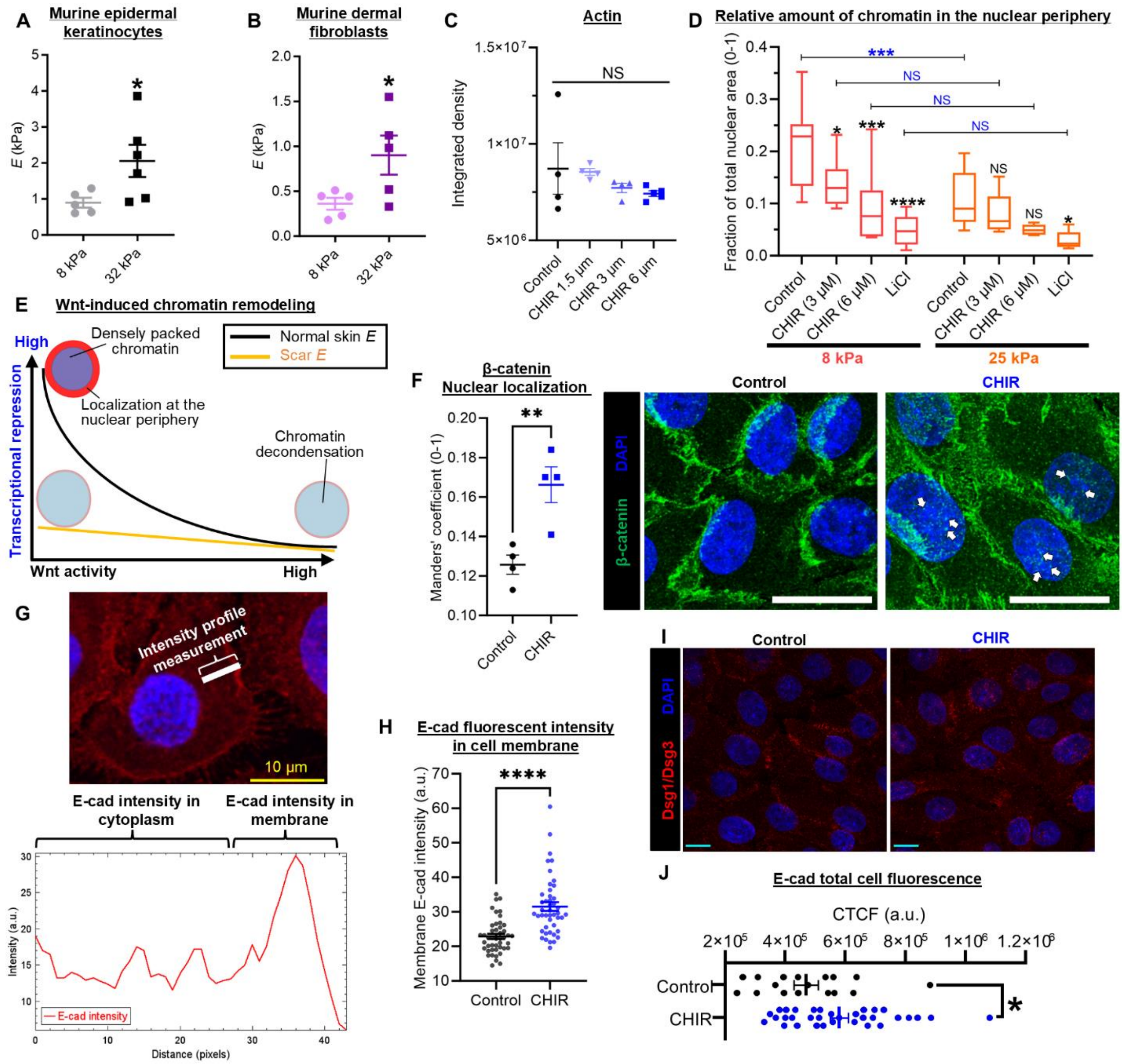

Fig. S4. Effect of canonical Wnt signaling on cellular mechanotransduction. (A and B) Normal substrate rigidity response in (**A**) untreated murine epidermal keratinocytes and (**B**) dermal fibroblasts cultured at substrate *E* (x-axis) of 8 or 32 kPa. Cell rigidity scaled with substrate rigidity for both cell types in these control groups. *n*=5-6 force maps per condition. (**C**) Integrated density of fluorescence for phalloidin staining in CHIR-treated human keratinocytes. *n*=4-5 fields per condition (12-23 cells/field). (**D**) Relative amount of chromatin in the nuclear periphery. Represented as aggregate area of H2B localization clusters in the periphery, divided by total nuclear area. *n*=8 nuclei per condition. **(E)** Wnt-induced chromatin remodeling is blunted in a scar-like environment. In untreated keratinocytes, the transcriptional environment is more suppressive on normal skin *E* (more compact chromatin nanodomains and increased localization at the nuclear periphery) vs. those on scar *E*. On both substrate rigidity values, Wnt agonist treatment promotes chromatin decondensation to facilitate the transcription of Wnt target genes. However, more subtle changes in chromatin architecture induced by low levels of Wnt agonist are seen on normal skin *E* only. (**F**) CHIR enhances nuclear translocation of β-catenin

(dose=3 µM). Nuclear translocation was assessed by measuring the degree of colocalization between DAPI and β-catenin (Manders' coefficient: 0=no colocalization, 1=perfect colocalization) to reveal an increase in the CHIR-treated group. *n*=4 high-power fields per condition (10-20 cells/field). Nuclear β-catenin expression appreciable as discrete fluorescent foci (arrows) in CHIR-treated keratinocytes but not untreated controls. Scale, 20 µm. (**G and H**) CHIR enhances E-cadherin expression on the cell membrane. (**G**) To measure E-cadherin expression on the cell membrane via image analysis, the fluorescence intensity profile (bottom) of a line drawn between the cytoplasm and the cell membrane (top) was captured and analyzed. From intensity measurements (expressed as arbitrary unit, a.u.) taken from pixel regions corresponding to the cell membrane, the peak intensity value was calculated to serve as a marker of membrane E-cadherin expression. Scale, 10 µm. (**H**) Membrane E-cadherin expression became significantly elevated after CHIR treatment for 48 hours. *n*=45 cell-cell junctions (15 cell-cell junctions measured from 3 separate fields for each group). (**I**) Representative confocal images of desmogleins 1/3 expression (red) in CHIR-treated human keratinocytes. Desmoglein expression was unaltered after CHIR treatment. Scale, 20 µm. (**J**) Total cell fluorescence measurements of E-cadherin were significantly higher in CHIR-treated cells than in control cells. *n*=17-33 cells from 3-4 separate fields for each group.

Data represented as mean ± s.e.m. or box and whisker plots (median, Q1 and Q3). $^*P < 0.05$; $^{**}P < 0.01$; $^{***}P < 0.001$; $^{****}P < 0.0001$.

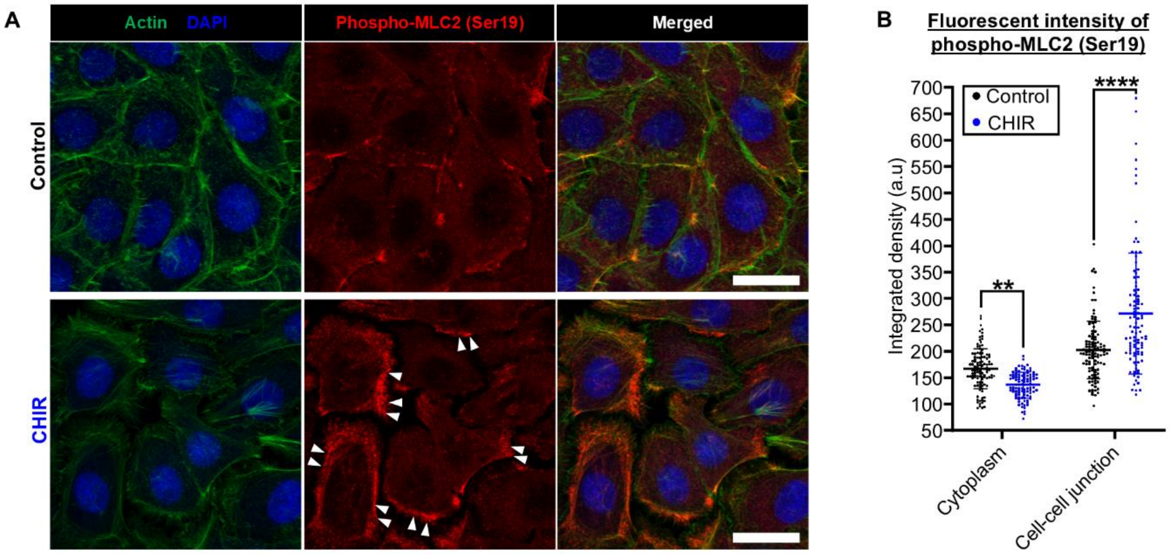

Fig. S5. Wnt signaling enhances phosphorylation of MLC2 at cell-cell junctions but not the cytoplasm. Expression of phosphorylated MLC2 (phospho-MLC2) at serine 19 residue in human epidermal keratinocytes after 48 hours of treatment with CHIR (6 µM). **(A)** Representative confocal images with staining for actin (phalloidin staining, green) and phospho-MLC2 (red) with DAPI (blue) as the nuclear counterstain. In CHIR-treated cells, note the elevated phospho-MLC2 expression at cell-cell junctions (white arrowheads). Phalloidin staining of CHIR-treated cells showed actin network resembling a "hollowed-out" appearance (*i.e.*, sparse in the center and rich in the periphery). Scale, 20 µm. **(B)** Fluorescent intensity of phospho-MLC2 at the cytoplasm and the cell-cell junction. In CHIR-treated cells, the integrated density of fluorescence for phospho-MLC2 staining was lower in the cytoplasm and higher in cell-cell junctions. n=100-110 cells measured from 3 separate dishes for each condition. Data represented as mean ± s.d. $^{**}P < 0.01$, $^{****}P < 0.0001$.

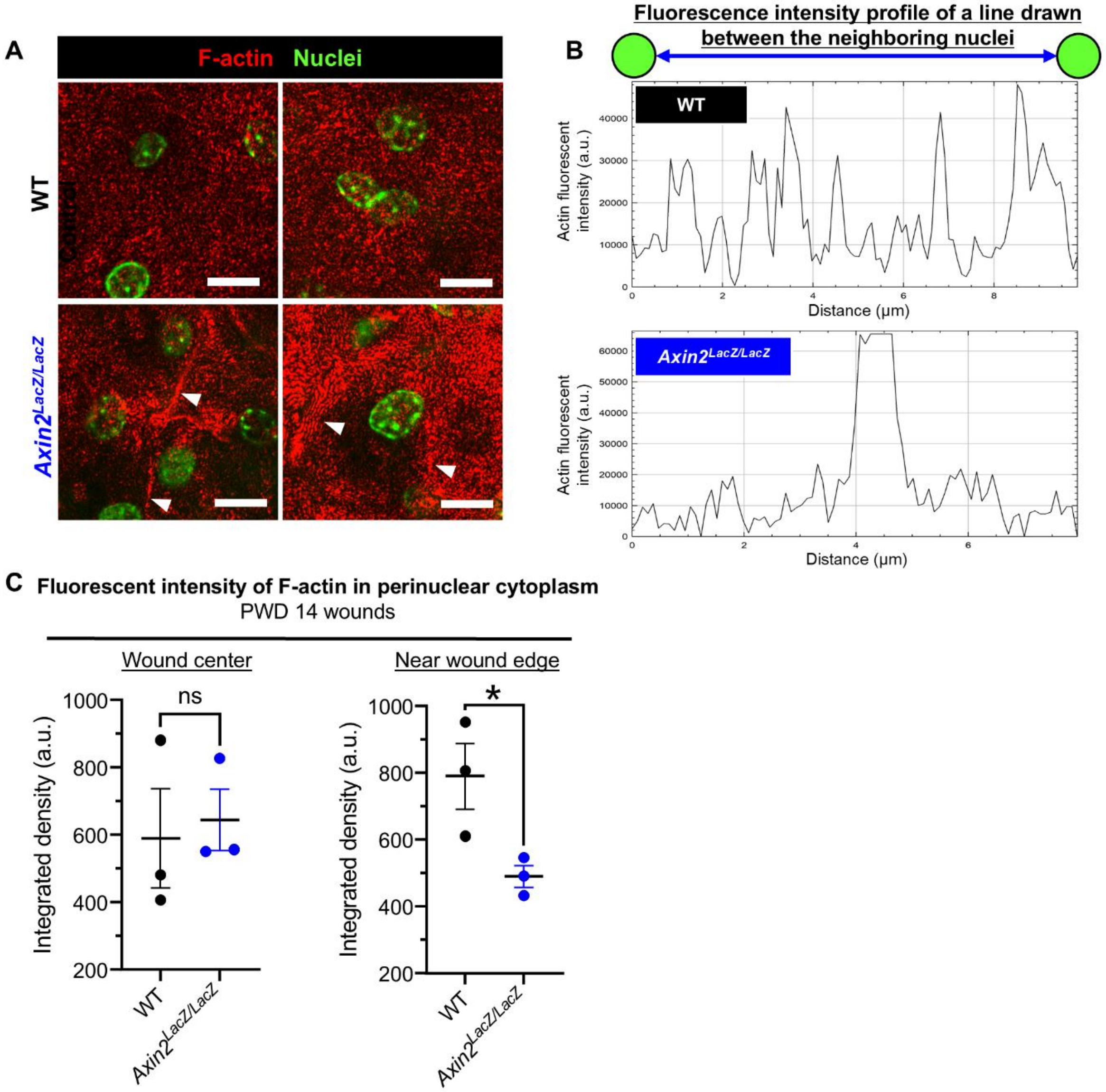

Fig. S6. Live-cell imaging of the actin network in wound tissue harvested on PWD 14. (A) Representative confocal images of the intracellular actin network from the healed wound epidermis of WT and *Axin2*$^{LacZ/LacZ}$ mice. *Axin2*$^{LacZ/LacZ}$ mice demonstrated actin architecture that closely mirrored the "hollowed-out" appearance (*i.e.*, sparse in the center and rich in the periphery, white arrows) seen in CHIR-treated keratinocytes *in vitro*. Scale, 10 µm. (**B**) Representative fluorescence intensity profile of actin along a line drawn between neighboring nuclei for WT and *Axin2*$^{LacZ/LacZ}$ mice. **(C)** Between the inner 2/3 of the wound and the wound edge (*i.e.*, near wound edge), the fluorescence intensity of the actin network was significantly lower in the *Axin2*$^{LacZ/LacZ}$ group—in line with cytoskeletal changes associated with lower tissue rigidity. *n*=3 mice per group. Each point represents the mean of intensity measurements from 25-52 cells in 2-4 Z-levels of an image stack captured from 1 animal (blinded analysis performed using Filename_Randomizer macro on ImageJ). Data represented as mean ± s.e.m. *P < 0.05.

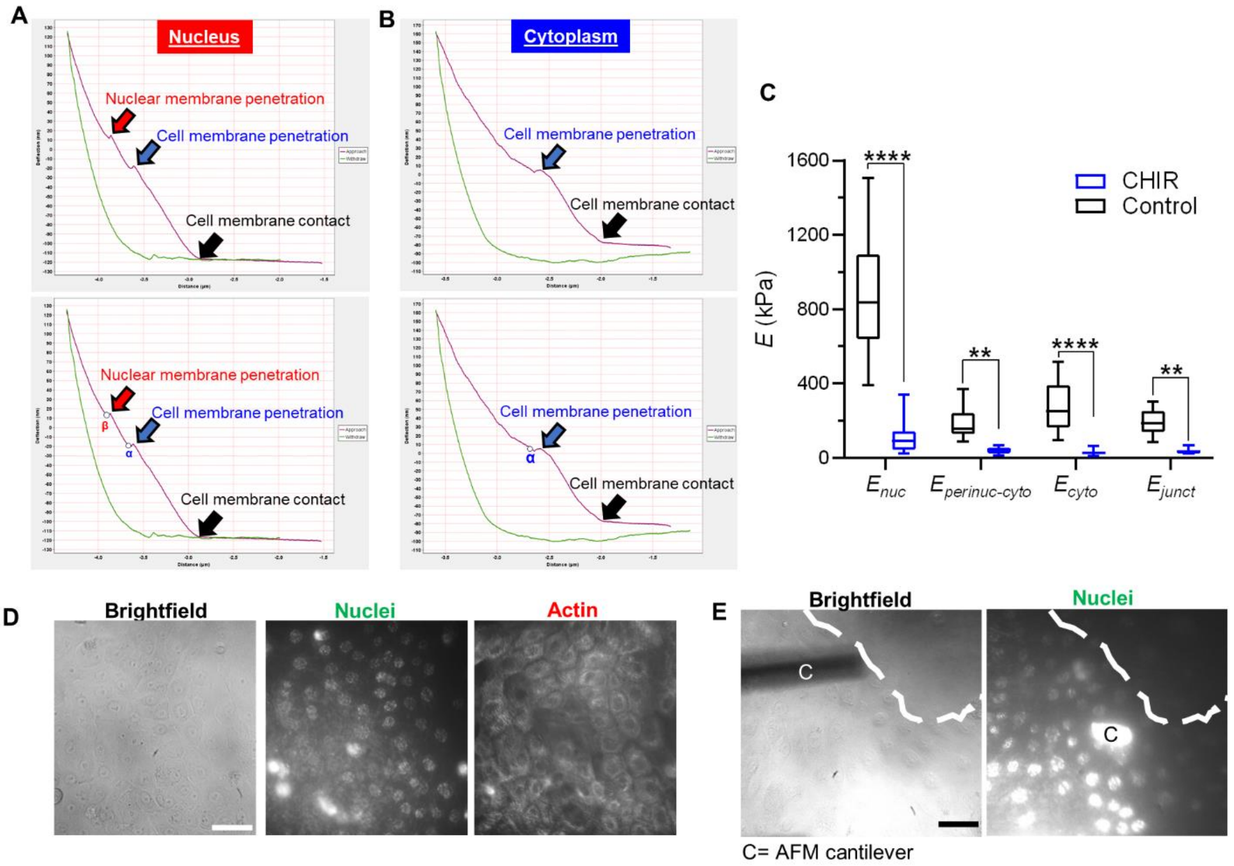

Fig. S7. Nanoneedle AFM analysis of live human keratinocytes. (**A**) A representative force-distance curve from nuclear measurement. The purple line represents the approach phase of AFM, and the green line represents the withdrawal phase. Two successive local force peaks during the approach phase were used to identify sites of cell membrane penetration (point α) and nuclear membrane penetration (point β). The contact model for a cone tip was fitted between the cell membrane contact point and point α to calculate *E* of the perinuclear cytoplasm. To calculate *E* of the nucleus, the contact model was fitted between points α and β. (**B**) A representative force-distance curve from cytoplasmic measurement. A local force peak was used to identify the site of cell membrane penetration (point α). The contact model for a cone tip was fitted between the cell membrane contact point and point α to calculate *E*. (**C**) Intracellular compartments of living cells were directly probed by a nanoneedle to obtain AFM measurements used to calculate *E*. Human keratinocytes cultured on a glass surface. n=15-17 cell measurements per group. (**D**) Site selection for nanoneedle AFM, guided by live-cell imaging. Brightfield microscopy, paired with fluorescent microscopy using nuclear and actin dyes, enabled direct visualization of intracellular structures for targeted measurements. Scale, 50 µm. (**E**) Direct visualization of the monolayer's edge (dotted line) during nanoneedle AFM. The cantilever (labeled C) was placed over regions of interest for measurements. Scale, 50 µm.

Data represented as mean ± s.e.m. or box and whisker plots (median, Q1 and Q3). $^{**}P < 0.01$; $^{****}P < 0.0001$.

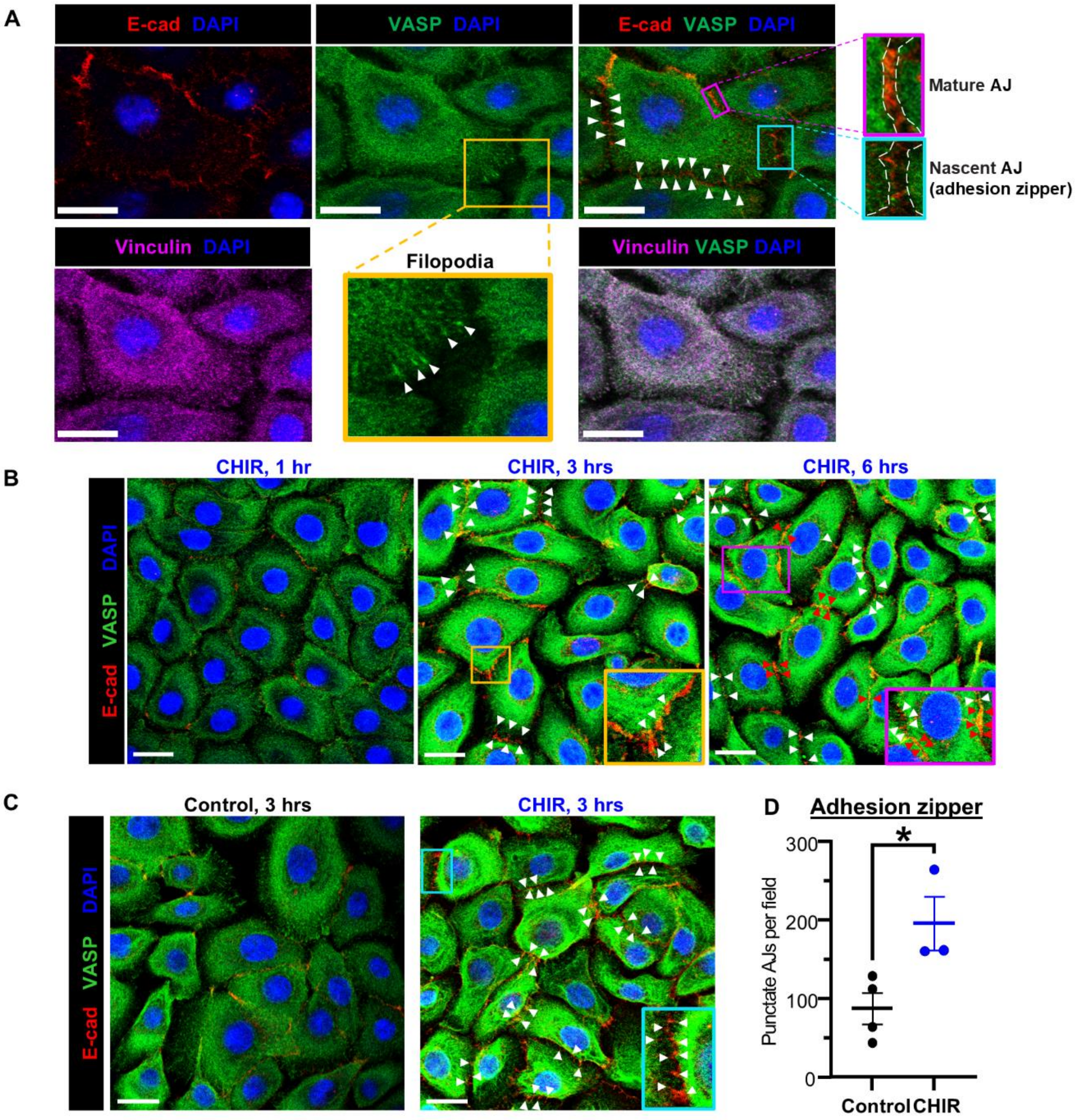

Fig. S8. Wnt signaling triggers the formation of new AJs. (**A**) Adhesion zipper formation in untreated human epidermal keratinocytes harvested 12-24 hours after plating. Representative confocal images of adhesion zipper components, E-cadherin (red), VASP (green) and vinculin (magenta), are shown. Initially, filopodia (finger-like protrusions from the membrane, yellow inset on bottom middle) form adhesive contacts between neighboring cells. Nascent AJs emerge as puncta (punctate AJs, white arrowheads) arranged as two rows (*i.e.*, on each side of the membrane) to form the adhesion zippers (cyan inset, top right). The adhesion zippers, which interlock together to form a mature AJ with linear morphology (magenta inset, top right), contain copious amounts of actin filaments and actin regulators, including VASP and vinculin. Scale, 20 µm. (**B-D**) Wnt triggers the formation of adhesion zippers. (**B**) The timing of adhesion zipper formation in CHIR-treated keratinocytes. Cells were harvested after CHIR treatment for 1, 3, or 6 hours. For each timepoint, representative confocal images with staining for E-cadherin (red) and VASP (green) are shown. Adhesion zippers, composed of rows of E-cadherin+, VASP+ puncta (white arrowheads), emerged

~1-3 hours after treatment (middle panel). After 6 hours, some adhesion zippers began to merge to form mature AJs with linear morphology (red arrowheads). The magenta inset shows a cell with partially merged adhesion zippers (white arrowheads) with linear AJ morphology seen on inferior side only (red arrowheads). Scale, 20 µm. (**C**) Representative images of cells after 3 hours of CHIR treatment (right) vs. those from the untreated control group (left). E-cadherin (red) and VASP (green) staining are shown. Adhesion zippers (white arrowheads) were much more prevalent in the CHIR-treated group. Scale, 20 µm. (**D**) Quantitative analysis of adhesion zippers after 3 hours of treatment with CHIR. The number of E-cadherin+, VASP+ puncta (*i.e.,* punctate AJs) was counted for each high-power field (*n*=3-4 fields per group. 20-30 cells/field) to reveal a significant increase in the CHIR-treated group. Data represented as mean $\pm$ s.e.m. *$P < 0.05$.

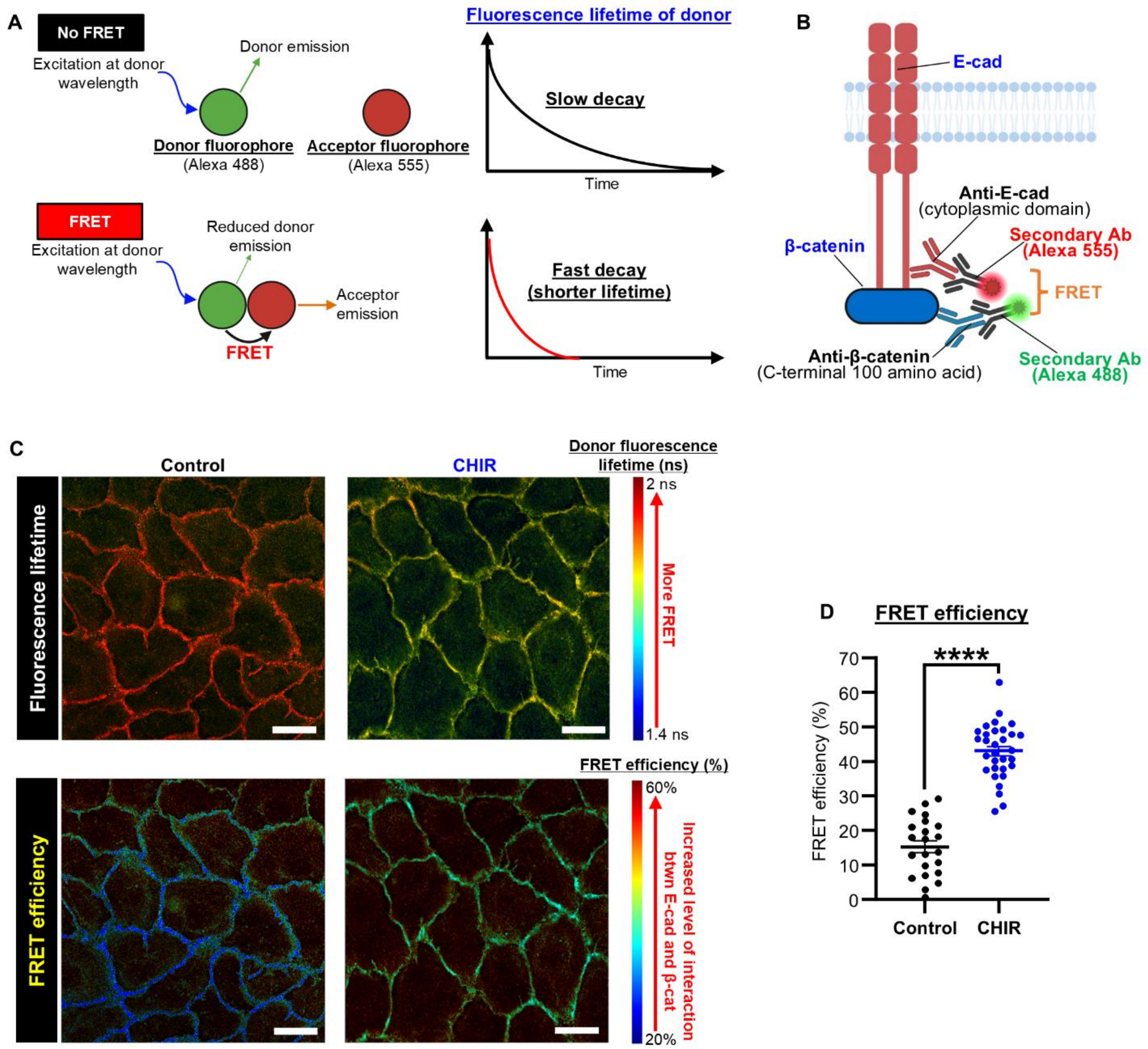

Fig. S9. FLIM-FRET analysis of protein-protein interactions between E-cadherin and β-catenin on the cell membrane. (**A**) Measurement of protein-protein interactions using FLIM-FRET. Two proteins are fluorescently labeled as a donor-acceptor pair (*e.g.*, Alexa 488 and Alexa 555) with significant spectral overlap (*i.e.*, emission spectrum of the donor fluorophore overlaps with absorption spectrum of the acceptor fluorophore). The donor fluorophore absorbs photons emitted by the light source at donor wavelength to reach an excited state. The donor fluorophore then undergoes fluorescence decay by emitting photons, and the donor fluorescence lifetime is then measured. FRET, or nonradiative transfer of energy from the donor to the acceptor fluorophore, occurs when the two fluorophores are in close proximity (less than 10 nm apart). This transfer of energy during FRET accelerates the rate of fluorescent decay and shortens the donor fluorescence lifetime. FRET efficiency is calculated by comparing the average lifetime of the donor with and without the acceptor. The FRET efficiency increases with stronger protein-protein interactions (*i.e.*, binding events), lower intermolecular distance between the donor-acceptor pair and favorable conformational changes that facilitate the energy transfer event. (**B**) Fluorescent labeling of E-cadherin and β-catenin during FLIM-FRET. Following overnight incubation using antibodies targeting E-cadherin (cytoplasmic domain)

vs. β-catenin (C-terminal 100 residues, part of the interaction surface between E-cadherin and β-catenin (*1*)), fluorescent-dye conjugated secondary antibodies were added to fluorescently label β-catenin (FRET donor, Alexa 488) and E-cadherin (FRET acceptor, Alexa 555). (**C**) Representative fluorescence lifetime images of human keratinocytes demonstrating a significant reduction in the donor fluorescence lifetime in the CHIR-treated group. The shorter donor fluorescence lifetime seen in CHIR-treated cells indicates increased FRET. Scale, 20 μm. (**D**) Wnt agonist treatment increases the FRET efficiency at cell-cell junctions. Human keratinocytes were treated with CHIR for 48 hours prior to FLIM-FRET. FRET efficiency measurements were obtained from cell-cell junctions (n=22-31 per group, taken from 3-4 separate fields for each group). In both untreated and CHIR-treated groups, appropriate donor-only control samples were included in the analysis—which showed nearly identical donor fluorescence lifetime (2.97 ns) in both groups. Data represented as mean ± s.e.m. ****$P < 0.0001$.

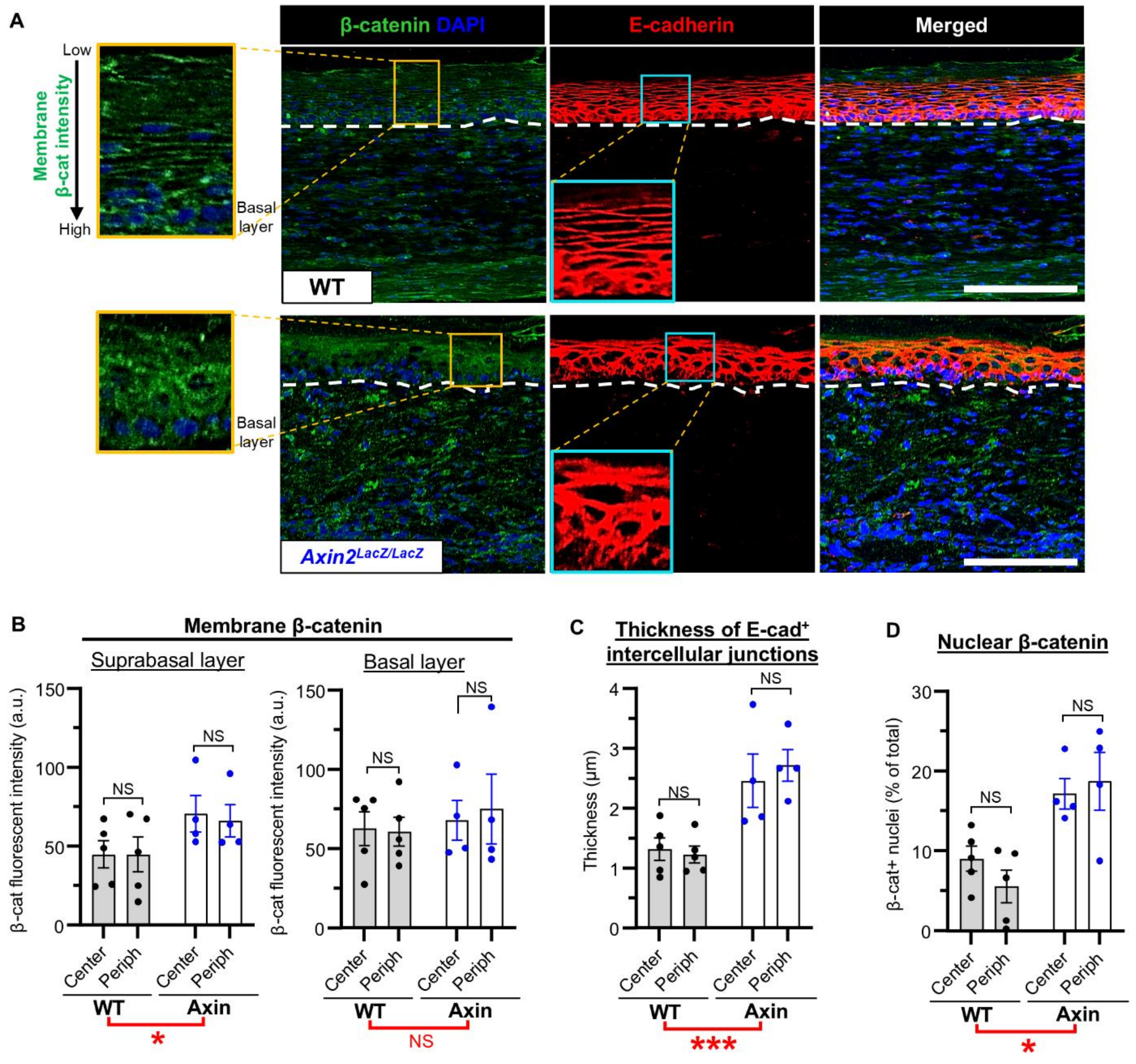

Fig. S10. Membrane expression of β-catenin is amplified at the suprabasal epidermis of wounds in *Axin2*$^{LacZ/LacZ}$ mice. β-catenin and E-cadherin expressions from the wound center in WT and *Axin2*$^{LacZ/LacZ}$ mice harvested on PWD 14. (**A**) Representative confocal images of β-catenin (green) and E-cadherin (red) expression. Basement membrane depicted as a dashed white line. In WT mice, β-catenin staining showed intense membrane expression on the basal but not the suprabasal layer of the epidermis (inset with yellow border, top left). In *Axin2*$^{LacZ/LacZ}$ mice, however, β-catenin was robustly expressed in both the basal and the suprabasal layers (inset with yellow border, top left). Similar findings were observed for E-cadherin staining with *Axin2*$^{LacZ/LacZ}$ mice showing thicker E-cadherin+ intercellular junctions (inset with cyan border, bottom of middle panel). Scale, 100 μm. (**B**) Fluorescence intensity of β-catenin in cell-cell junctions of the wound epidermis. Compared to WT controls, *Axin2*$^{LacZ/LacZ}$ mice showed significantly higher intensity measurements at the suprabasal layer (left). The basal layer (right) showed comparable intensity levels in both groups. *n*=4-5 mice per group. (**C**) Thickness of E-cadherin+ cell-cell junctions between keratinocytes in the wound epidermis with higher thickness measurements seen in the *Axin2*$^{LacZ/LacZ}$ group. *n*=4-5 mice per group. (**D**)

Nuclear expression of β-catenin. The number of β-catenin+ nuclei was significantly higher in the *Axin2*$^{LacZ/LacZ}$ group. *n*=4-5 mice per group. Data represented as mean ± s.e.m. **P* < 0.05; ****P* < 0.001.

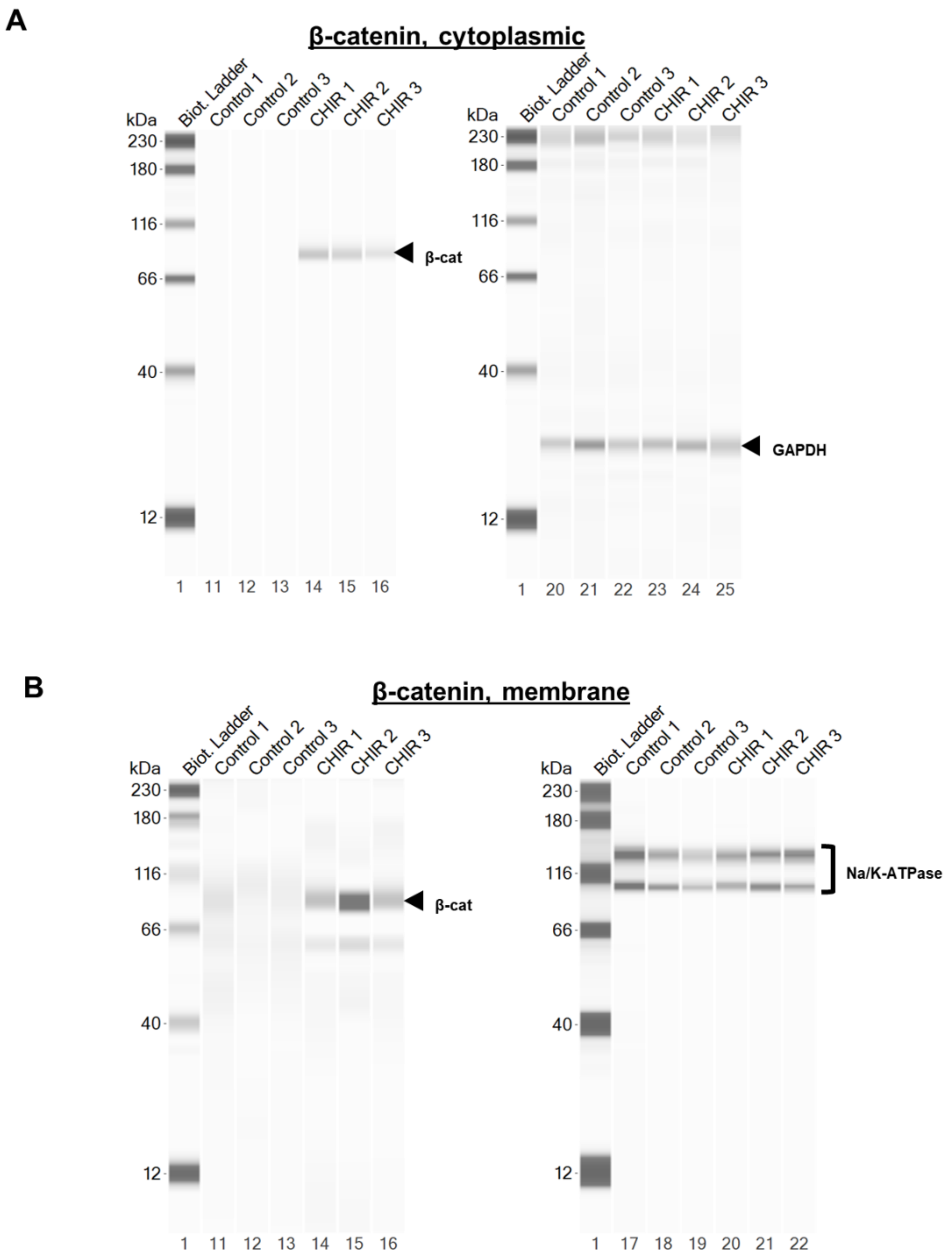

Fig. S11. Unprocessed Simple Western blot images. (**A**) Simple Western immunoblots of cytoplasmic β-catenin (92 kDa) and GAPDH (36 kDa). (**B**) Simple Western immunoblots of membrane β-catenin and Na/K-ATPase. Note 2 bands for Na/K-ATPase that correspond to MWs of an intact enzyme (150 kDa) and its α1 subunit (100 kDa). Area measurements for 2 bands were averaged during the analysis step.

CENTER		Mean	SEM	Min	Max	*p*-value†
**Collagen fiber length (um)**	WT	30.7	0.9	29.2	32.9	0.23
	AXIN	33.1	1.5	29.4	38.2	
**Collagen fiber width (um)**	WT	2.3	0.1	2.1	2.5	0.67
	AXIN	2.4	0.1	2.2	2.5	
**Collagen fiber density (fibers per 100 um^2)**	WT	0.61	0.03	0.54	0.67	0.71
	AXIN	0.59	0.02	0.53	0.67	
**Coherency of collagen network (0 to 1)***	WT	0.32	0.03	0.25	0.38	0.13
	AXIN	0.44	0.06	0.25	0.56	
INNER 1/3		Mean	SEM	Min	Max	*p*-value†
**Collagen fiber length (um)**	WT	30.1	1	27.8	32.6	0.09
	AXIN	32.2	0.6	30.4	33.8	
**Collagen fiber width (um)**	WT	2.3	0.1	2.1	2.4	0.07
	AXIN	2.5	0.1	2.3	2.7	
**Collagen fiber density (fibers per 100 um^2)**	WT	0.62	0.03	0.57	0.7	0.63
	AXIN	0.64	0.01	0.59	0.67	
**Coherency of collagen network (0 to 1)***	WT	0.33	0.07	0.19	0.52	0.16
	AXIN	0.43	0.02	0.36	0.49	
INNER 2/3		Mean	SEM	Min	Max	*p*-value†
**Collagen fiber length (um)**	WT	30.9	0.7	29.4	32.3	0.26
	AXIN	32	0.7	29.9	33.6	
**Collagen fiber width (um)**	WT	2.4	0.1	2.3	2.6	0.47
	AXIN	2.5	0.1	2.3	2.6	
**Collagen fiber density (fibers per 100 um^2)**	WT	0.6	0.03	0.53	0.68	0.26
	AXIN	0.64	0.02	0.58	0.67	
**Coherency of collagen network (0 to 1)***	WT	0.4	0.03	0.36	0.48	0.95
	AXIN	0.4	0.03	0.28	0.46	
PERIPHERY		Mean	SEM	Min	Max	*p*-value†
**Collagen fiber length (um)**	WT	30.3	0.9	27.8	31.9	0.26
	AXIN	30.3	0.3	29.5	30.8	
**Collagen fiber width (um)**	WT	2.6	0.1	2.3	2.7	0.47
	AXIN	2.6	0	2.5	2.6	
**Collagen fiber density (fibers per 100 um^2)**	WT	0.65	0.03	0.6	0.71	0.26
	AXIN	0.66	0.01	0.63	0.7	
**Coherency of collagen network (0 to 1)***	WT	0.34	0.04	0.27	0.42	0.95
	AXIN	0.35	0.02	0.3	0.41	

*1= perfect alignment, 0=isotropic conditions. †Statistical analysis performed for collagen properties on each site using unpaired *t*-test between WT control group (*n*=4 animals) and *Axin2*$^{LacZ/LacZ}$ group (*n*=5 animals).

Table S1. **Collagen properties during WIHN on the day of scab detachment**. SHG measurements obtained from tissue sections of wounds.

Target	Oligo sequence (5'-3')
Human *CDH1*	Forward: CGAGAGCTACACGTTCACGG<br>Reverse: GGGTGTCGAGGGAAAAATAGG
Human *CDH3*	Forward: ATCATCGTGACCGACCAGAAT<br>Reverse: GACTCCCTCTAAGACACTCCC
Human *GAPDH*	Forward: ACAACTTTGGTATCGTGGAAGG<br>Reverse: GCCATCACGCCACAGTTTC
Mouse *Gadph*	Forward: AGGTCGGTGTGAACGGATTTG<br>Reverse: TGTAGACCATGTAGTTGAGGTCA
Human *LEF1*	Forward: AGAACACCCCGATGACGGA<br>Reverse: GGCATCATTATGTACCCGGAAT
Mouse *Lef1*	Forward: TGTTTATCCCATCACGGGTGG<br>Reverse: CATGGAAGTGTCGCCTGACAG
Human *PXN*	Forward: CTGCTGGAACTGAACGCTGTA<br>Reverse: GGGGCTGTTAGTCTCTGGGA
Human *TLN1*	Forward: TACATGCTCCGAAATGGGGAC<br>Reverse: ACCATGATCGTCTTCACAGTTC

Table S2. List of primer sequences used for qPCR analysis.

Movie S1: Microfabrication of a nanoneedle using dual-beam plasma focused ion beam milling (time-lapse video, 20 frames per second). Serial scanning electron microscopy images of the AFM probe were captured before, during and after the milling.
